# Splicing factor USP39 promotes ovarian cancer malignancy through maintaining efficient splicing of oncogenic HMGA2

**DOI:** 10.1038/s41419-021-03581-3

**Published:** 2021-03-17

**Authors:** Shourong Wang, Zixiang Wang, Jieyin Li, Junchao Qin, Jianping Song, Yingwei Li, Ling Zhao, Xiyu Zhang, Haiyang Guo, Changshun Shao, Beihua Kong, Zhaojian Liu

**Affiliations:** 1 Department of Obstetrics and Gynecology, Qilu Hospital, Cheeloo College of Medicine, Shandong University, 107 Wenhua Xi Road, Jinan, 250012 Shandong Province China; 2 Key Laboratory of Experimental Teratology, Ministry of Education, Department of Cell Biology, Cheeloo College of Medicine, Shandong University, Jinan, Shandong 250012 China; 3 Molecular Medicine and Genetics, Cheeloo College of Medicine, Shandong University School of Medicine, 44 Wenhua Xi Road, Jinan, Shandong 250012 China; 4 Department of Clinical Laboratory, The Second Hospital of Shandong University, Jinan, 250012 China; 5 Institutes for Translational Medicine, State Key Laboratory of Radiation Medicine and Protection, Soochow University, Suzhou, 215123 Jiangsu Province China

**Keywords:** RNA, Gynaecological cancer

## Abstract

Aberrant expression of splicing factors was found to promote tumorigenesis and the development of human malignant tumors. Nevertheless, the underlying mechanisms and functional relevance remain elusive. We here show that USP39, a component of the spliceosome, is frequently overexpressed in high-grade serous ovarian carcinoma (HGSOC) and that an elevated level of USP39 is associated with a poor prognosis. USP39 promotes proliferation/invasion in vitro and tumor growth in vivo. Importantly, USP39 was transcriptionally activated by the oncogene protein c-MYC in ovarian cancer cells. We further demonstrated that USP39 colocalizes with spliceosome components in nuclear speckles. Transcriptomic analysis revealed that USP39 deletion led to globally impaired splicing that is characterized by skipped exons and overrepresentation of introns and intergenic regions. Furthermore, RNA immunoprecipitation sequencing showed that USP39 preferentially binds to exon-intron regions near 5′ and 3′ splicing sites. In particular, USP39 facilitates efficient splicing of HMGA2 and thereby increases the malignancy of ovarian cancer cells. Taken together, our results indicate that USP39 functions as an oncogenic splicing factor in ovarian cancer and represents a potential target for ovarian cancer therapy.

## Introduction

More than 95% of human genes undergo alternative splicing^1^, which allows individual genes to produce multiple RNA and protein isoforms. Alternative splicing has also been found to be associated with various diseases including human cancers^2^. Aberrant splicing can lead to loss-of-function of tumor suppressors or activation of oncogenes and cancer pathways^3^. Recurrent mutation of splicing factors is observed in a variety of malignancies, and SF3B1, U2AF1, and SRSF2 are the three most frequently mutated splicing factors^4^. Mutations in the RNA splicing factor SF3B1 promote tumorigenesis through c-MYC stabilization^5^. Altered expression of splicing factors such as SRSF1 was found to cause changes in hundreds of alternative splicing events in multiple cancer types^6,7^.

Ovarian cancer is the most lethal gynecologic malignancy and the overall 5-year survival rate is around 30%^8^. Ovarian cancer is usually associated with advanced stage at diagnosis, lymph node metastasis, and resistance to chemotherapy^9^. Aberrant expression of splicing factors have been implicated in the development of ovarian cancer. Splicing factor SRp20 is required for ovarian tumor cell growth and maintenance of transformation properties^10^. Overexpression of ESRP1 predicts poor prognosis and promotes epithelial-mesenchymal transition (EMT) via alternative splicing of CD44 and ENAH^11^. Our previous study found that splicing factor CTNNBL1 is upregulated in ovarian cancer cells and that an elevated level of CTNNBL1 indicates poor prognosis in patients with high-grade serous ovarian carcinoma (HGSOC)^12^. However, it remains a challenge to identify the hub splicing factors and relevant alternative splicing events in ovarian cancer development.

Here, using a quantitative proteomic approach to compare protein accumulation in HGSOCs and noncancerous tissue, we found that proteins upregulated in HGSOCs were significantly enriched in spliceosome pathway proteins. Of these proteins, splicing factor USP39 was frequently overexpressed in HGSOCs and associated with a poor prognosis. We next identified USP39-regulated alternative splicing events by performing RNA-sequencing (RNA-seq) and RNA immunoprecipitation sequencing (RIP-seq). We verified that USP39 is required for the efficient splicing of HMGA2 (high-mobility group AT-hook 2). Our study highlights the biological significance of USP39 in ovarian cancer development and identifies USP39-modulated splicing events relevant to ovarian cancer pathogenesis.

## Materials and methods

### Patients and tissue samples

Ovarian cancer and FT tissues were collected at the Department of Obstetrics and Gynecology, Qilu Hospital, Shandong University from 2006 to 2016. Malignant tumor specimens were from primary ovarian cancer patients with no previous surgery or chemotherapy treatment. FT tissues were from patients who underwent a total hysterectomy and bilateral salpingo-oophorectomy for uterine diseases or benign neoplastic adnexal pathologic changes. All participants were given written informed consent, and the Ethics Committee of Shandong University approved the study (SDULCLL2019-1-09). Information about the clinical samples used in this study is supplied in Supplementary Table 1.

### iTRAQ labeling coupled with LC-MS/MS analysis

Proteomics analysis of 10 samples of HGSOC and FT tissues was conducted at PTM-BioLab according to standard procedures. In brief, after trypsin digestion, peptides were dried by vacuum centrifugation. Peptides were then reconstituted and processed according to the manufacturer’s protocol for iTRAQ labeling (Applied Biosystems). LC-MS/MS analysis was then conducted as previously described^13^. We set 1.5-fold change as the different expression threshold and *p* < 0.05 as the significance threshold. Proteins enriched in HGSOCs are shown in Supplementary Table 2.

### Plasmid constructs and transfection

HA-USP39 and HA-GFP retrovirus plasmids were from Addgene (#22581 and #22612). The USP39 lentivirus plasmid with 3 × FLAG tags was from Genechem. The shUSP39 sequences (sh1 and sh2) were cloned into the pGIPZ vector (Open Biosystem). HMGA2 minigenes were cloned into the pCDNA3 vector (Invitrogen). The c-MYC expression plasmid was from Genechem. The sh-c-MYC sequence was cloned into pLKO.1 vector (Addgene). The retrovirus vectors were transfected into ΦNX packaging cells to produce retroviral particles. The lentivirus vectors were transfected with psPAX2 and pMD2.G into HEK293T cells to produce lentiviral particles. Stable cell lines were established by retrovirus or lentivirus infection followed by puromycin (2 µg/ml) selection for two weeks. USP39 small interfering RNA(siRNA) was synthesized by RiboBio. Plasmids and siRNAs were transfected into cells using jetPRIME transfection reagent (PolyPlus). RNA and protein were extracted 48 or 72 h after transfection. The sequence of shRNAs and siRNAs are shown in Supplementary Table 3.

### Cell lines and cell culture

Human ovarian cancer cell lines A2780, SKOV3, OVCAR3, OVCAR8, and CAOV3 were purchased from American Type Culture Collection. Mouse ovarian cancer cell line ID8 was from Sigma-Aldrich. HEY and HEK293T cells were obtained from Jian-Jun Wei’s lab. A2780, OVCAR8, CAOV3, HEY, ID8, Hela, and HEK293T cells were cultured in DMEM (Gibco). SKOV3 was cultured in McCoy’s5A medium. The medium was supplemented with 10% fetal bovine serum (Gibco). The cell lines were confirmed by unique short tandem repeat analyses and mycoplasma testing.

### Colony formation assay

A2780 and OVCAR8 cells with USP39 overexpression or knockdown were seeded into six-well plates (400 cells per well). After 1–2 weeks of incubation, cells were fixed with methanol and stained with 0.1% crystal violet (Solarbio). Quantification was achieved by counting the number of cell colonies. The data were presented as the mean ± SD and represented three independent experiments.

### Cell Counting Kit-8 (CCK-8) assay

Cells were seeded in 96-well plates (800 cells per well) and incubated with 10 µl CCK-8 solution per well for 4 h at 37 °C. The absorbance at 450 nm was quantified by a microplate spectrophotometer (BioRad). Cell proliferation rate was represented as the ratio of absorbance over five days to absorbance in the first day. All experiments were performed in triplicate.

### EdU assay

Cells were seeded in 24-well plates (5 × 10^4^ cells per well) and cultured overnight. The EdU assay was performed using the EdU Kit (RiboBio, catalog no. C10310-1) according to the manufacturer’s instructions. In brief, cells were incubated in a culture medium containing 1:1000 diluted EdU for 30 min. Then cells were fixed and stained by Apollo fluorescent dye and Hoechst. Images were captured from three random fields under a fluorescent microscope.

### Matrigel invasion assay

The matrigel invasion assay was performed in 24-well chambers with 8 μm pores (BD Biosciences) coated with matrigel (Corning). A cell suspension containing 1 × 10^5^ cells in serum-free culture medium (200 µl) was plated into the upper chambers and 700 µl culture medium containing 30% fetal bovine serum was added to the lower chambers. The chambers were incubated at 37 °C for 12–36 h. Cells that migrated through the membrane were fixed with methanol and stained with 0.1% crystal violet. The number of invaded cells was counted under a light microscope.

### Nude mice xenografts

Female BALB/c-nude mice (female, 4–5-week old; Charles River) were randomly divided into the indicated groups (5–10 mice of each group) and subjected to subcutaneous or intraperitoneal injection with luciferase-expressing ovarian cancer cells with USP39 overexpression or knockdown. Bioluminescence images were captured using an imaging system (PerkinElmer). The Shandong University Animal Ethics Research Board approved all animal procedures.

### RNA isolation and qPCR

Total RNA from tissues or cells was extracted with TRIzol reagent (Invitrogen) according to the manufacturer’s protocol. RNA was reverse transcribed into cDNA using the HiScript II Q RT SuperMix for qPCR Kit (Vazyme). qPCR was performed with SYBR Green mix and the Bio-Rad CFX96. GAPDH served as an endogenous control. The 2^−ΔΔCT^ method was used for the relative quantification strategy of qRT-PCR data. Primer sequences are listed in Supplementary Table 3.

### Western blot

Cells were lysed on ice with lysis buffer (Sangon Biotech). Protein concentration was determined with the BCA Protein Assay Kit (Beyotime Institute of Biotechnology). Protein samples were separated by SDS-PAGE and electro-transferred to PVDF membrane. The membrane was blocked by incubating in 5% skimmed milk for 1 h and incubated overnight with primary antibodies at 4 ˚C. Proteins of interest were detected with HRP-labeled secondary antibodies and the ECL system (GE Healthcare). GAPDH was used as an endogenous control. All antibody information is listed in Supplementary Table 4.

### Immunohistochemistry staining

IHC staining of paraffin-embedded tissue microarray (TMA) sections was conducted with an immunohistochemistry staining kit (ZSGB-BIO) following the manufacturer’s instructions. In brief, tissue slides were deparaffinized in xylene and rehydrated in a graded series of ethanol. Antigen retrieval was performed in EDTA by heating in a microwave. Tissue slides were blocked with 1.5% normal goat serum and then incubated with primary antibody overnight at 4 °C. Then the slides were incubated with secondary antibody and stained with diaminobenzidine. The final IHC staining score for each sample was determined by two pathologists in a blinded manner based on the intensity and extent of staining across the section. The intensity of staining was scored as 0 (negative), 1 (weak), 2 (moderate), and 3 (strong). The extent of staining was based on the percentage of positive tumor cells: 1 (0–25%), 2 (26–50%), 3 (51–75%), and 4 (76–100%). The final IHC staining score was generated by multiplying the percentage score with the staining intensity score. Each sample had two replicates, and the final score was the average score of the replicates. Each sample was scored as low expression if the final score was less than or equal to 7 and high expression if the final score was greater than 7.

### Co-IP and MS

A2780 cells with exogenously expressed FLAG-tagged USP39 were harvested, and cell pellets were lysed on ice with Western and IP Cell Lysis Buffer (Beyotime Institute of Biotechnology). Whole-cell extracts were incubated with 5 µg FLAG antibodies (CST) for 1 h, followed by incubation with magnetic Protein A/G beads (Bimake) overnight at 4 °C. Beads were washed with Western and IP Cell lysis Buffer three times, and immunocomplex was resuspended in 1× SDS-PAGE loading buffer and separated by SDS-PAGE followed by Coomassie brilliant blue staining. The peptides were detected by tandemMS and confirmed by western blotting.

### RNA-seq and data analysis

Total RNA was isolated from USP39 knock-down and controlled A2780 cells (three replications of each sample) using TRIzol reagent (Invitrogen) according to the manufacturer’s protocol and then next-generation sequencing was performed by the Ribobio Biotechnology Company. The RNA-seq data generated in this study have been deposited in the NCBI GEO database under the accession number GSE157365. Log2 fold change >1 or <−1 and *p* < 0.05 was the threshold for different expression.

### RNA immunoprecipitation sequencing

A2780 cells with ectopically expressed FLAG-tagged USP39 were collected for the RIP assay, which was performed using the EZ-Nuclear RIP (Cross-Linked) Kit (Merck Millipore) following the manufacturer’s instructions. In brief, cells were cross-linked with 0.3% formaldehyde. Nuclei were extracted and lysed in nuclei lysis buffer, and DNA was sheared to about 500–1000 bp fragments by sonication. Nuclei lysates were incubated with magnetic beads coated with anti-FLAG antibody (Cell Signaling Technology). Then RNA was extracted from immunocomplexes and digested by DNaseI. Both input and RIP samples were prepared for next-generation sequencing by the Ribobio Biotechnology Company. The RIP-seq data generated in this study has been deposited in the NCBI GEO database under the accession number GSE157401.

### Luciferase assay

USP39 wild-type and mutant promoter sequences were cloned into separate pGL4.26 plasmids (Promega). Then USP39 promoter reporter vector was co-transfected into HEK293T cells with the pRL-TK and c-MYC plasmids. Luciferase activity was measured 48 h after transfection using the Dual-Luciferase Reporter Assay System (Promega). The relative luciferase activity was calculated as the ratio of firefly luminescence and Renilla luminescence.

### Splicing reporter assay

The splicing reporter assays were conducted as previously described^14^. In brief, plasmids encoding luciferase with or without an intervening intron were co-transfected with USP39 (si-USP39) or negative control small interfering RNA (si-NC) into Hela cells. The pRL-TK vector was used as a control. Luciferase activity was detected 48 h after transfection using the Dual-Luciferase Reporter Assay System (Promega). Data were normalized for transfection efficiency by calculating the ratio between firefly and Renilla luciferase activity.

### RNA pull-down assay

HMGA2 transcripts were obtained using the RNAMAX-T7 in vitro transcription kit (RiboBio) and then biotin-labeled using the Pierce™ RNA 3′ End Desthiobiotinylation Kit (Thermo Fisher Scientific). RNA pull-down was performed using the Magnetic RNA-Protein Pull-Down Kit (Thermo Fisher Scientific) according to the manufacturer’s protocol. The proteins were detected by western blot analysis.

### Immunofluorescence

A2780 cells were fixed with 4% paraformaldehyde for 30min at room temperature followed by permeabilization with 0.5% Triton X-100 in PBS for 15 min at room temperature. Samples were then blocked with 1% BSA for 1 h at room temperature and incubated with primary antibody overnight at 4 °C (SC35,1:500; USP39,1:200). Samples were washed with PBS containing 0.1% Triton X-100 and incubated with secondary antibody for 1 h at room temperature. The images were captured by an Andor Revolution confocal microscope system.

### Bioinformatics analysis

GO analysis was performed with KOBAS3.0 software. GO provides label classification of gene function and gene product attributes (http://www.geneontology.org). KEGG pathway analysis was conducted using KOBAS3.0 software (http://www.genome.jp/kegg). The pre-RNA splicing efficiency was analyzed according to the previously reported workflow in yeast^15^. In brief, reads were aligned to the hg19 genome with HISAT2 (version 2.2.0) and sorted with samtools (version 1.9). Regtools 0.2.0 and Bedtools (version 2.27.1) were used to identify putative splicing events and transreads. Splicing efficiency was determined separately for the 5′and 3′ splice site as follows: Efficiency 5′ = transread count/5′ intron end first base coverage; Efficiency 3′ = transread count/3′ intron end last base coverage. R/R Bioconductor 3.6.3 was used for plotting. Based on the crosslinked RIP-seq data, reads were trimmed and aligned to the hg19 genome with HISAT2 (version 2.2.0) and sorted with samtools (version 1.9). All the intron-exon junction and exon-intron junction sites detected in the FLAG-USP39 and input samples were identified with Regtools (version 0.2.0) and converted to bed files. Then all the sequences were mapped to the region around the junction (–100 to 100 bp). Deeptools was used for plotting the curve of reads distribution and density. In order to detect the RIP binding peak distribution, raw reads were trimmed and known rRNA sequences were removed. Tophat (version 2.0.13) was used to align clean reads to the hg19 genome. RIP peaks were identified and annotated by RIPSeeker, an R package based on the Hidden Markov Model. Further analysis and plotting were performed using R/R Bioconductor 3.6.3. Kaplan–Meier plotter (http://kmplot.com/analysis/) was used to analyze the association of USP39 and HMGA2 mRNA expression with overall survival and progression-free survival of patients with HGSOCs.

### Statistical analysis

Student’s *t*-test and one-way ANOVA analysis was used to determine significance. The chi-square test was used to analyze the differences in clinical characteristics. The log-rank test was used to detect differences in clinical prognosis. Results represent the mean ± SD of three independent experiments. The threshold for statistical significance was *P* < 0.05.

## Results

### USP39 is commonly upregulated in HGSOCs and correlates with poor prognosis

**Fig. 1:**
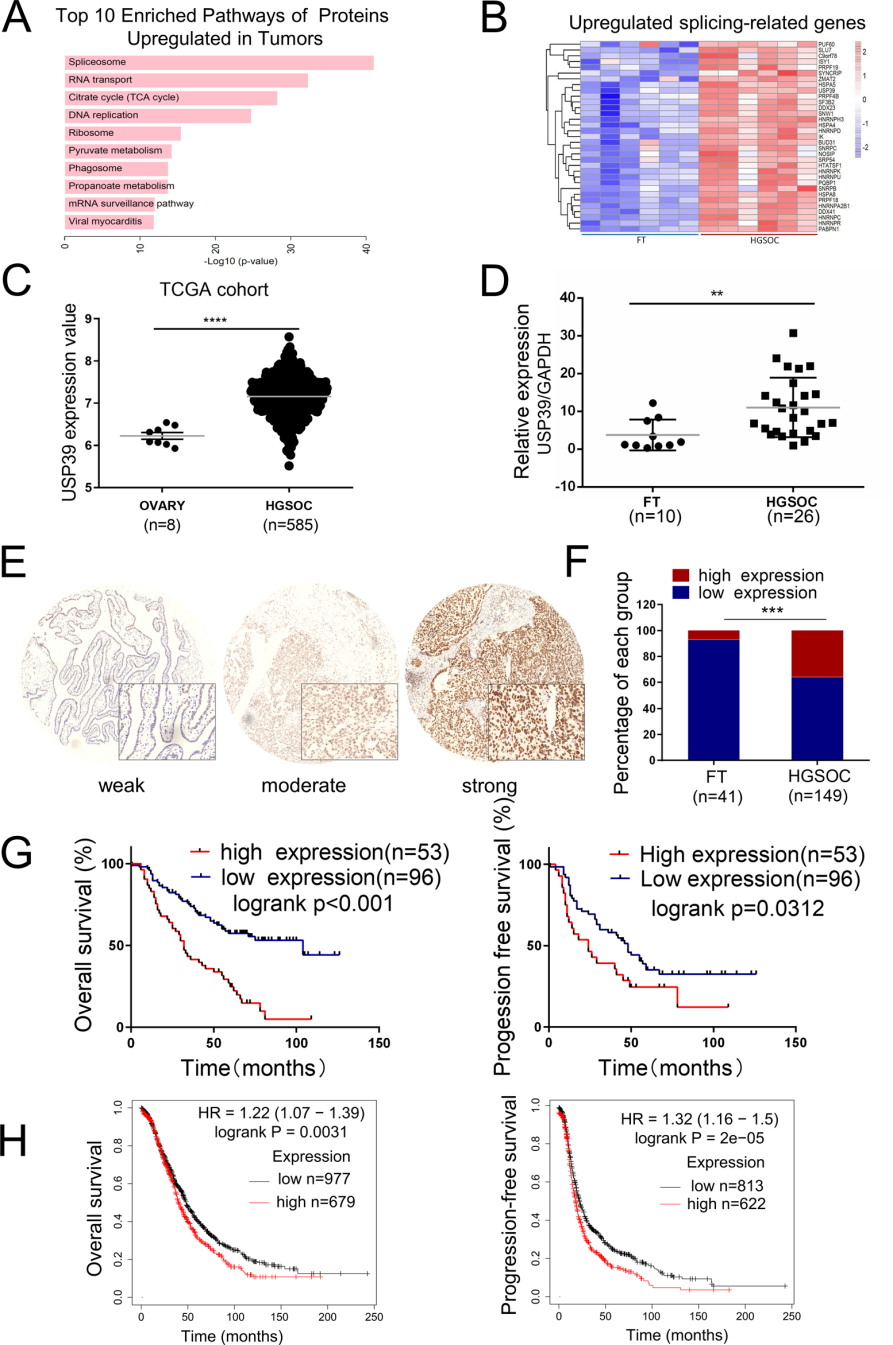
USP39 is frequently upregulated in HGSOCs and correlates with poor prognosis. **A** Pathway analysis of proteins enriched in HGSOCs (*n* = 10) compared with FTs (*n* = 10) based on proteomics data. Samples were collected from Qilu Hospital, Shandong University. **B** Heat map showing expression levels of splicing factors upregulated in HGSOCs (*n* = 6) compared with FTs (*n* = 6) based on transcriptome microarray results (GSE135886). Samples were collected from Qilu Hospital, Shandong University. **C**, **D** USP39 mRNA expression was compared between HGSOCs and normal tissues from the TCGA cohort (**C**) and our cohort (**D**). **E** Representative images of immunohistochemical (IHC) staining of USP39 in our tissue microarray (containing 149 samples of HGSOCs and 41 samples of FTs collected from Qilu Hospital, Shandong University). **F** Statistical analysis of USP39 expression from IHC staining of our tissue microarray. High and low expression indicate the final score of each sample determined by two pathologists based on the intensity and extent of staining across the tissue microarray section. HGSOC patients were dichotomized into low (Score ≤7) and high (Score >7) USP39 protein expression groups based on IHC staining score. **G** Kaplan–Meier analysis of the correlation between USP39 expression and clinical prognosis based on data from our tissue microarray. **H** Kaplan–Meier analysis of the effect of USP39 mRNA expression on the overall survival and progression-free survival of ovarian cancer patients based on an online cohort from Kaplan–Meier Plotter (http://kmplot.com/). The high and low expression groups were separated based on the best cutoff. *P* value was obtained by Student’s *t*-test (**C**, **D**, **F**) or Log-rank test (**G**, **H**). *P < 0.05, **P < 0.01, ***P < 0.001.

We performed quantitative proteomics analysis of HGSOCs (*n* = 10) and fallopian tube (FT) tissues (*n* = 10) using iTRAQ labeling and mass spectrometry (MS) (Supplementary Table 2). Pathway enrichment analysis showed that the spliceosome pathway was the most significantly enriched pathway (Fig. 1A). Therefore, we focused on the spliceosome pathway and performed transcriptome analysis on core splicing factors in HGSOCs and FTs (GSE135886). Interestingly, a quarter (33 of 134) of core splicing factors were upregulated in HGSOCs compared with FTs (Fig. 1B). USP39 was selected for further investigation since it was significantly highly expressed in HGSOCs at both the protein and RNA level. We further examined the expression level of USP39 in the TCGA (The Cancer Genome Atlas) cohort and found that it was more highly expressed in most HGSOC samples compared with normal ovary samples (Fig. 1C). In addition, we validated USP39 expression by qPCR and, as illustrated in Fig. 1D, it was frequently overexpressed in HGSOCs (*n* = 26) compared with FTs (*n* = 10). Importantly, pan-cancer analysis^16^ showed that USP39 was upregulated in various cancer types (Supplementary Fig. S1).

To evaluate the clinical significance of USP39 in HGSOC, we performed immunohistochemistry using a TMA containing 149 ovarian cancer tissue samples. As expected, immunohistochemical (IHC) staining revealed a significant increase of USP39 expression (Fig. 1E–F), and a high level of USP39 was correlated with advanced tumor stage and platinum resistance in HGSOC (Supplementary Table 5). We further assessed the prognostic value of USP39 in HGSOCs in our cohort of patients through Kaplan–Meier survival analysis, and found that a high level of USP39 was significantly correlated with poor overall survival and progression-free survival (Fig. 1G). The prognostic value of USP39 in HGSOCs was further verified using an online cohort from Kaplan–Meier plotter (http://kmplot.com/analysis/) (Fig. 1H). Taken together, these observations strongly indicate that upregulation of USP39 is closely associated with disease progression and poor prognosis in patients with HGSOC.

### USP39 increases proliferation/invasion of ovarian cancer cells and promotes growth of xenograft tumors in mice

**Fig. 2:**
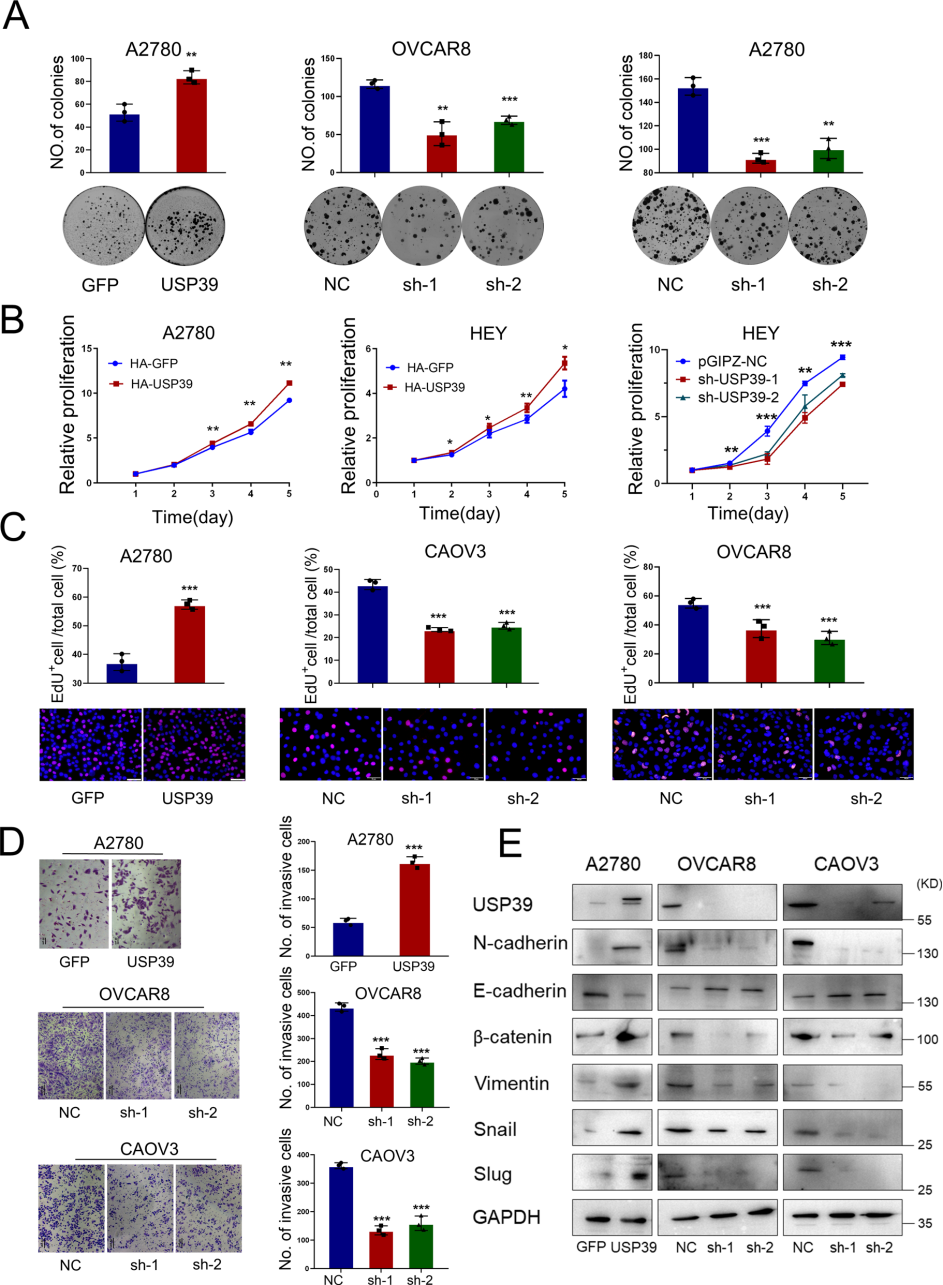
USP39 promotes proliferation/invasion and epithelial-mesenchymal transition (EMT) of ovarian cancer cells. **A**–**C** Effect of USP39 on the proliferation of ovarian cancer cells was examined by (**A**) clonogenic, (**B**) growth curve and (**C**) EdU assays of ovarian cancer cells with USP39 overexpression (USP39) or knock down (sh-1 and sh-2) compared to corresponding control (*n* = 5 for clonogenic assay and *n* = 3 for growth curve and EdU assays). The results of these assays indicated that overexpression of USP39 promoted, whereas knockdown of USP39 prevented, the proliferation of ovarian cancer cells. **D** Matrigel invasion data showing that overexpression of USP39 significantly enhanced invasion capacity, whereas knockdown of USP39 (sh-1 and sh-2) decreased the invasion potential of ovarian cancer cells (*n* = 3 biologically independent samples). **E** Abundance of EMT-related markers in ovarian cancer cells with USP39 overexpression or knockdown was measured by western blot analysis. *P* value was obtained by Student’s *t*-test. Results represent the mean ± SD of three independent experiments. *P < 0.05, **P < 0.01, ***P < 0.001.

We next explored whether USP39 was functionally relevant for ovarian progression. USP39 was overexpressed and knocked down in ovarian cancer cells using retrovirus and lentivirus infection, respectively. The overexpression and knockdown efficiency were detected by western blot (Supplementary Fig. S2). We then performed a clonogenic assay and found that USP39 dramatically promoted the clonogenic capacity, whereas USP39 inhibition led to reduced clonogenicity (Fig. 2A). Consistent with this, ectopic expression of USP39 also significantly enhanced the proliferation of ovarian cancer cells as measured by growth curve and EdU assays (Fig. 2B–C). In addition, a transwell invasion assay revealed that forced expression of USP39 significantly enhanced the invasion capacity whereas knockdown of USP39 decreased the invasion potential of ovarian cancer cells (Fig. 2D). Furthermore, we measured EMT markers by western blotting. Ectopic expression of USP39 led to upregulated expression of mesenchymal markers and reduced levels of epithelial markers in A2780 cells. On the contrary, USP39 knockdown resulted in decreased expression of mesenchymal markers and higher expression of epithelial markers in OVCAR8 and CAOV3 cells (Fig. 2E).

**Fig. 3:**
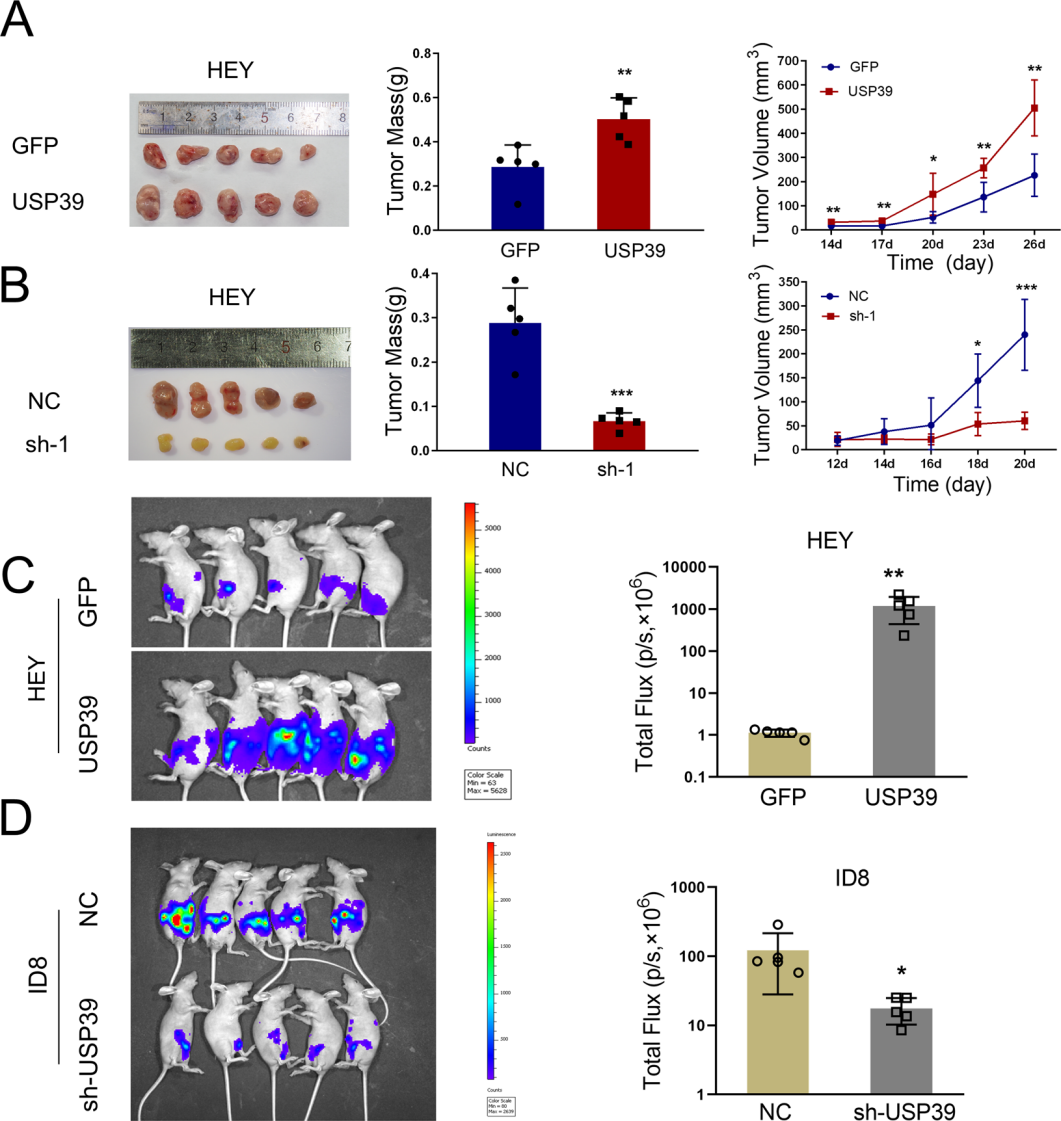
USP39 promotes tumorigenesis and progression of ovarian cancer in nude mice xenografts. **A**, **B** Images of xenograft tumors, tumor mass, and tumor volume in mice subcutaneously injected with HEY cells with USP39 (**A**) overexpression (USP39) or (**B**) knockdown (sh-USP39) compared to corresponding control (*n* = 5 mice per group). **C**, **D** Representative images of luciferase signals and quantification of photon flux in immunodeficient mice 4 weeks after intraperitoneal injection of luciferase-expressing (**C**) HEY and (**D**) ID8 cells with USP39 overexpression and knock down, respectively (*n* = 5 mice per group). *P* value was obtained by Student’s *t*-test. Results represent the mean ± SD. *P < 0.05, **P < 0.01, ***P < 0.001.

We further confirmed the function of USP39 in ovarian cancer tumorigenesis and progression in vivo. Firstly, HEY cells with USP39 overexpression or knock down were subcutaneously injected into nude mice (*n* = 5). As expected, forced expression of USP39 in HEY cells induced a significant increase in tumor mass and volume (Fig. 3A). USP39 depletion led to reduced tumor mass and volume (Fig. 3B). Next, we conducted intraperitoneal injection, which has been used to mimic human ovarian cancer growth. Luciferase-expressing ovarian cancer cells with USP39 overexpression or knockdown and control cells were intraperitoneally injected into nude mice (*n* = 5). Luciferase was used as a tracer for in vivo imaging analysis. The results showed that overexpression of USP39 in HEY cells led to an increased number of tumor nodes in the abdominal cavity (Fig. 3C). Consistent with this, mice injected with ID8 cells with USP39 knockdown had a significant reduction in tumor burden (Fig. 3D). Collectively, these findings supported the notion that USP39 exhibits oncogenic potential in the initiation and progression of ovarian cancer.

### c-MYC activates the transcription of USP39 in ovarian cancer cells

**Fig. 4:**
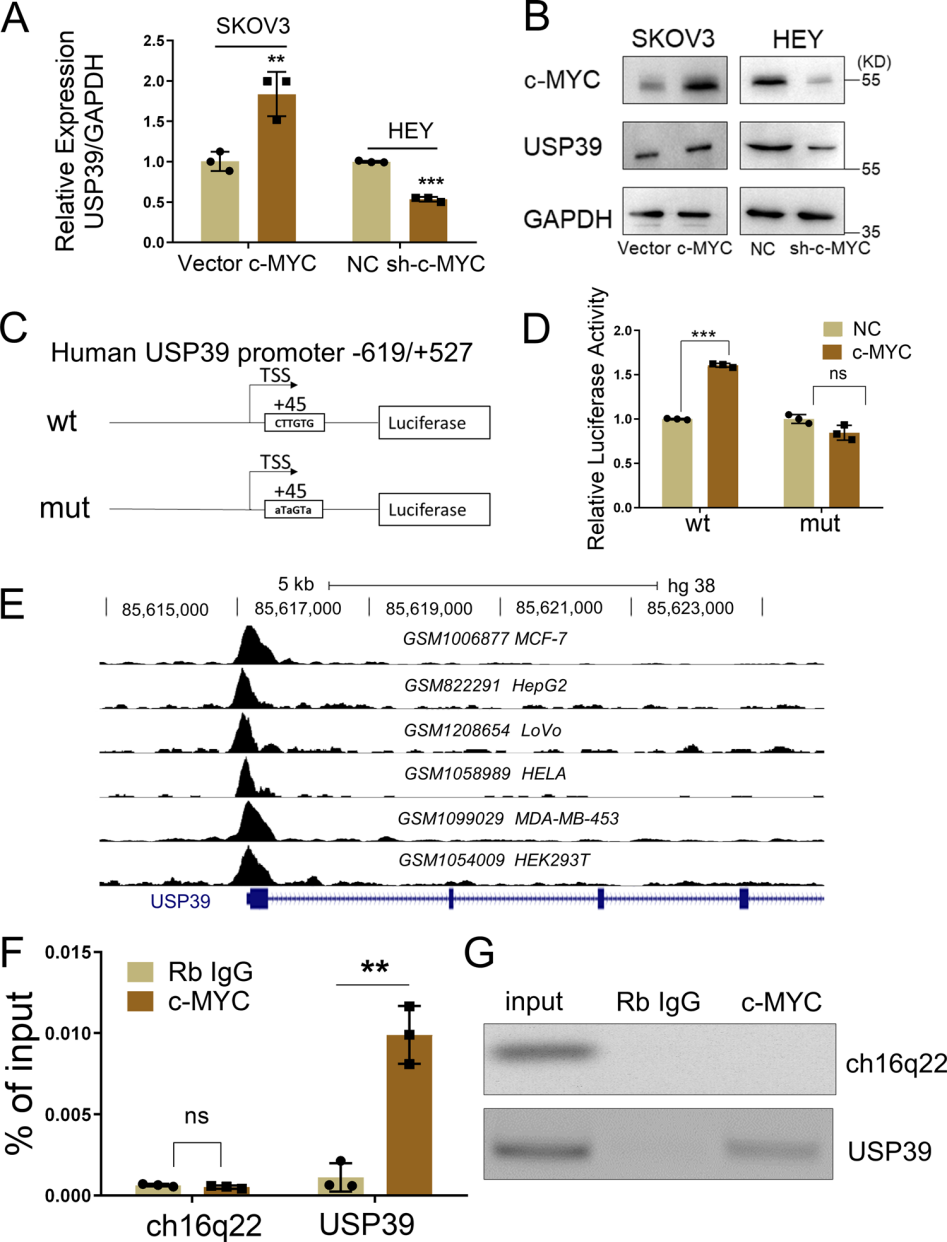
c-MYC activates the transcription of USP39 in ovarian cancer cells. **A** Relative expression of USP39 in ovarian cancer cells with c-MYC overexpression (c-MYC) or knock down (sh-c-MYC) compared to corresponding control (*n* = 3 biologically independent samples) was measured by qPCR. **B** Western blot analysis of c-MYC and USP39 protein levels upon c-MYC overexpression or knockdown. **C** Schematic diagram of the USP39 promoter (−619–+527) constructs based on the PGL4.26 vector. Wild type (wt) and Mutant (mut) refer to the wild-type and mutant versions of the predicted c-MYC binding site. **D** Luciferase activity was measured in HEK293T cells transfected with a c-MYC overexpression plasmid in combination with a pGL4 plasmid containing the wt or mut promoter region (*n* = 3 biologically independent samples). **E** Analysis of c-MYC binding peaks on USP39 promoter region in several cell lines based on ChIP-seq data in Cistrome Data Brower. **F**, **G** qPCR and semi-quantitative PCR analysis of ChIP samples from experiments performed in HEY cells using the c-MYC antibody or IgG. Ch16q22, an intergenic sequence, served as negative control. *P* value was obtained by Student’s *t*-test. Results represent the mean ± SD of three independent experiments. *P < 0.05, **P < 0.01, ***P < 0.001.

To explore potential factors that induced the ectopic expression of USP39 in ovarian cancer, we analyzed the USP39 promoter region using JASPAR (http://jaspar.genereg.net/). Interestingly, c-MYC was predicted to bind to the promoter region of USP39. We then measured USP39 expression in ovarian cancer cells with c-MYC overexpression or knockdown. As expected, overexpression of c-MYC significantly increased USP39 expression at both the RNA and protein level. In contrast, inhibition of c-MYC by shRNA decreased USP39 expression (Fig. 4A–B). The effect of c-MYC on USP39 expression was further investigated by performing a luciferase assay, and the result showed that forced expression of c-MYC increased luciferase activity of the USP39 promoter reporter containing the wild-type but not the mutant c-MYC binding site (Fig. 4C–D). We next analyzed ChIP-seq data from the Cistrome Data Browser database^17^ and found that sites bound by c-MYC were enriched in the promoter regions of USP39 in multiple cell lines (Fig. 4E), namely MCF-7, HepG2, LoVo, HElA, MDA-MB-453, and HEK293T. Next, we conducted a ChIP assay in HEY cells using a c-MYC antibody and found that c-MYC could directly bind to the USP39 promoter region (Fig. 4F–G). Taken together, these data suggested that USP39 is a direct transcriptional target of c-MYC in ovarian cancer cells.

### USP39 interacts with several spliceosome components and colocalizes with SC35 in nuclear speckles

**Fig. 5:**
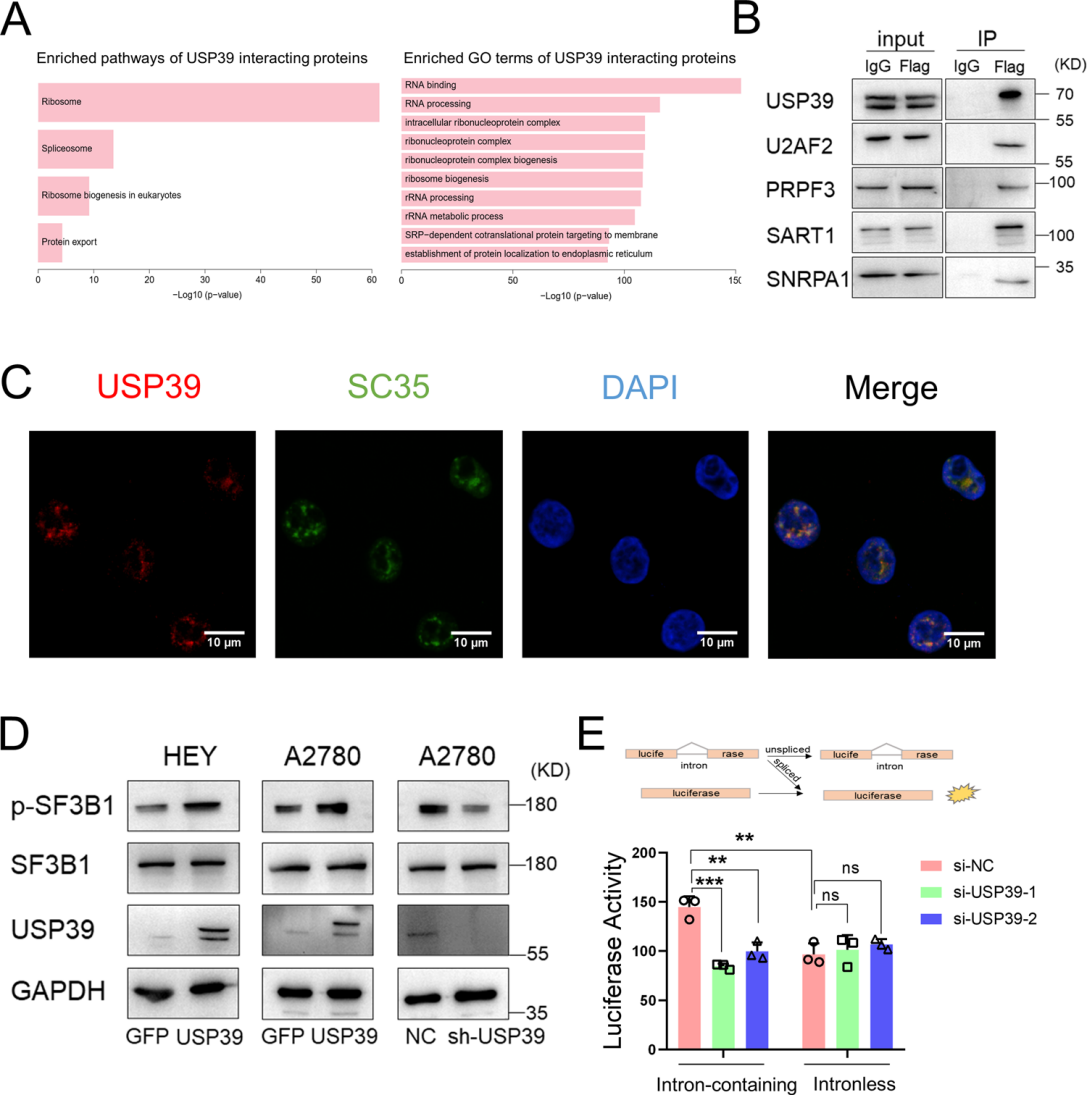
USP39 interacts with several spliceosome components and colocalizes with SC35 in nuclear speckles. **A** KEGG pathway and GO enrichment analysis of proteins pulled down by FLAG antibody from A2780 cells expressing FLAG-tagged USP39. **B** Co-IP of U2AF2, PRPF3, SART1, and SNRPA1 from A2780 cells expressing FLAG-tagged USP39 was detected by western blot. IgG was used as a negative control. **C** Immunofluorescence experiments revealed colocalization of USP39 (red) and splicing factor SC35 (green) in punctate nuclear speckles. **D** Western blot analysis of phospho-SF3B1(Thr313) and non-phosphorylated SF3B1 in HEY and A2780 cells with USP39 overexpression (HA-USP39) and control (HA-GFP) and A2780 cells with USP39 knock-down (sh-USP39) and control (pGIPZ-NC). **E** A splicing reporter assay was performed to assess the efficiency of splicing regulated by USP39. Inhibition of USP39 by small interfering RNAs (si-USP39-1 and si-USP39-2) significantly reduced the luciferase activity of an enzyme encoded by an intron-containing gene but had little effect on that encoded by an intronless gene (*n* = 3 biologically independent samples). *P* value was obtained by Student’s *t*-test. Results represent the mean ± SD of three independent experiments. *P < 0.05, **P < 0.01, ***P < 0.001.

USP39 was identified as a component of the U4/U6-U5 tri-snRNP complex in yeast^18^ and was found to regulate splicing in zebrafish^19^. The role of mammalian USP39 in the regulation of splicing remains unclear. To investigate the importance of USP39 in splicing regulation in ovarian cancer cells, FLAG-tagged USP39 was expressed in A2780 cells, then immunoprecipitated with an anti-FLAG antibody. Immunoprecipitated proteins were subjected to MS analysis, and the result showed that 28 of 134 core spliceosomal components were associated with USP39 (Supplementary Fig. S3 and Supplementary Table 6). Kyoto Encyclopedia of Genes and Genomes (KEGG) and gene ontology (GO) enrichment analysis also revealed that pathways related to the spliceosome, RNA binding, and RNA processing were significantly enriched among proteins interacting with USP39 (Fig. 5A). Next, we validated well-known splicing factors that potentially interact with USP39 based on MS data by performing coimmunoprecipitation (co-IP) and western blotting. SART1, U2AF2, and PRPF3 were verified as USP39-interacting partners (Fig. 5B).

Nuclear speckle is a type of nuclear body involved in splicing factor storage and modification^20^. Proteins assembled in nuclear speckles including PRPF3 and U2AF2 are known to modulate pre-mRNA processing^21,22^. To determine whether USP39 localizes at nuclear speckle, double-label immunofluorescence was performed with anti-USP39 and anti-SC35 (a marker for nuclear speckles). Intriguingly, we observed USP39 staining overlapped with SC35 (Fig. 5C), indicating that USP39 localizes at nuclear speckles and regulates splicing activity. SF3B1 is a splicing factor that lies at the catalytic core of the spliceosome and phosphorylation of SF3B1 is coupled with splicing catalysis^23^. To test whether USP39 modulate spliceosome activity via SF3B1, we measured phosphorylated SF3B1 in ovarian cancer cells with USP39 overexpression or knockdown. As a result, phosphorylated SF3B1 was upregulated in ovarian cancer cells with USP39 overexpression and was downregulated in those with USP39 depletion (Fig. 5D). In addition, a splicing reporter assay was implemented to explore the effect of USP39 on splicing. As demonstrated in Fig. 5E, small RNA interference of USP39 significantly reduced the activity of luciferase encoded by an intron-containing gene while it had little effect on that encoded by an intronless gene, implicating USP39 in splicing. These findings suggest that USP39 interacts with spliceosome components and may participate in pre-mRNA splicing.

### USP39 deletion induces global changes in alternative splicing patterns

**Fig. 6:**
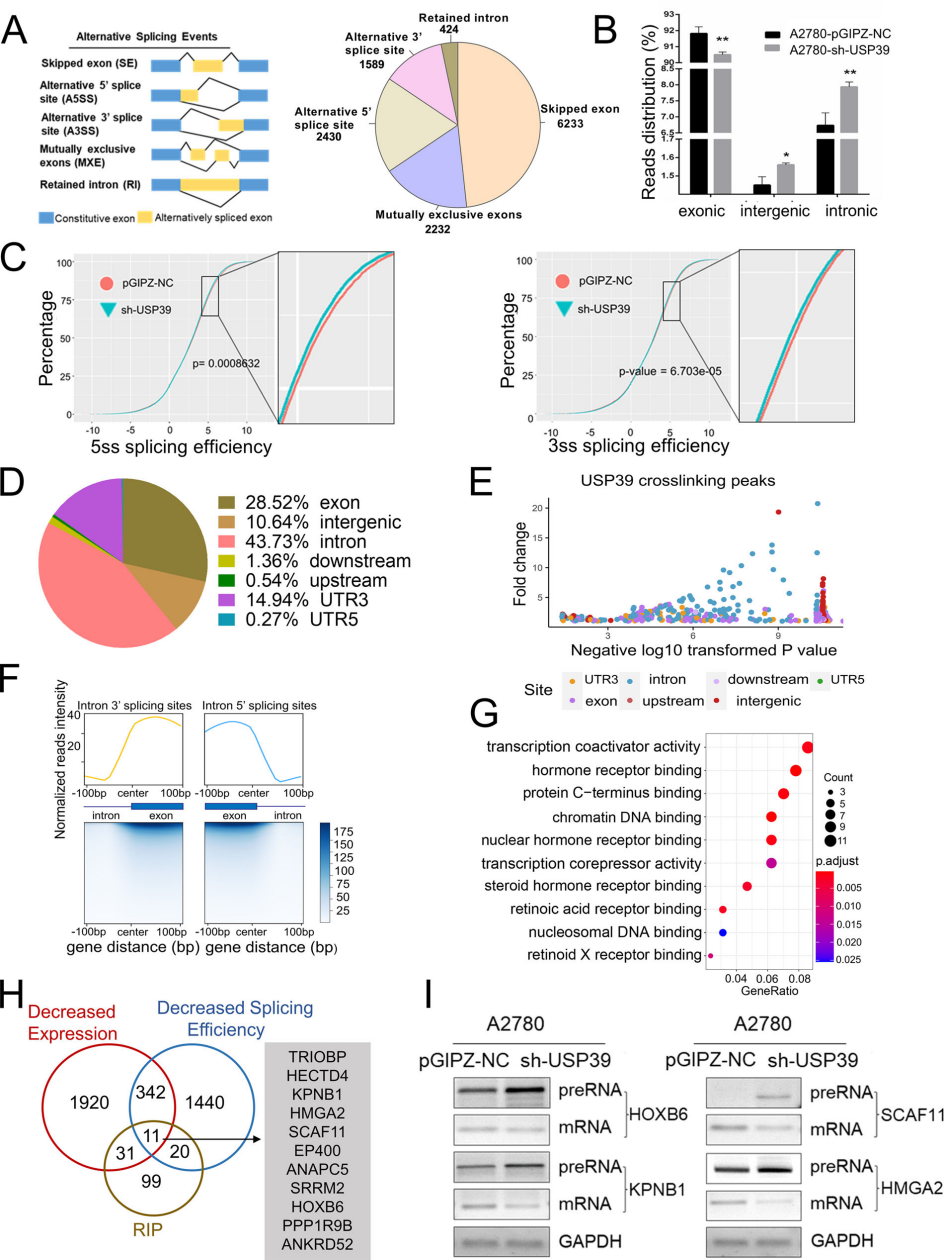
Identification of USP39-regulated splicing events by RNA-seq and RIP-seq. **A** Schematic diagram of alternative splicing types (left) and pie chart depicting the proportions of different types of alternative splicing events in A2780 cells response to loss of USP39 (right). **B** Distribution of the reads count over intronic, intergenic, and exonic regions in A2780 cells with USP39 knockdown and control cells (*n* = 3 biologically independent samples). **C** Global splicing efficiency analysis at 5′ splicing site (5ss) and 3′ splicing site (3ss) in A2780 cells with and without USP39 depletion by shRNA. **D** Pie chart of the distribution of USP39 binding peaks based on RIP-seq data from A2780 cells ectopically expressing FLAG-tagged USP39. Most peaks mapped to introns (43.73%) and exons (28.52%). **E** Distribution of USP39 binding peaks recognized by RIPSeeker (eFDR < 0.05) and their fold enrichment (from RIP-seq). A large proportion of the peaks with higher fold change values are located at intron and exon regions, indicating the potential effects of USP39 on pre-mRNA splicing. **F** Normalized read distribution around the 3′ and 5′ splicing sites (−100–100 bp) was analyzed using RIP-seq data from A2780 cells with ectopic FLAG-tagged USP39 expression. RIP-seq signal exhibited a bimodal distribution through the intron-exon-intron model, and most signal distributed around the intron-exon and exon-intron junction, indicating the assistant role of USP39 in intron-exon splicing and exons junction. **G** Bubble diagram showing the results of GO enrichment analysis of 161 genes encoding transcripts bound by USP39 (eFDR < 0.05) in A2780 cells with FLAG-USP39 overexpression (from RIP-seq). **H** The overlap in transcripts identified as being bound by USP39 (from RIP-seq) and transcripts with decreased expression and decreased splicing efficiency upon USP39 knockdown (from RNA-seq). **I** Semi-quantitative PCR validation of selected genes from (**F**). *P* value was obtained by Student’s *t*-test. Results represent the mean ± SD of three independent experiments. *P < 0.05, **P < 0.01, ***P < 0.001.

We next determined whether depletion of USP39 could alter the splicing pattern globally. We conducted RNA-seq on USP39 knockdown and wild-type A2780 cells (GSE157365). Alternative splicing analysis was performed using the rMATS software (http://rnaseqmats.sourceforge.net/index.html). We found that USP39 knockdown resulted in alterations in 12908 splicing events, including 6233 skipped exons, 2232 mutually exclusive exons, 2430 alternative 5′ splice sites, 1589 alternative 3′ splice sites and 424 retained introns (Fig. 6A). Analysis of the reads mapping distribution revealed that USP39 depletion significantly reduced the proportion of exonic reads, whereas it increased the proportion of intergenic and intronic reads (Fig. 6B), indicating that there is a widespread impairment in pre-mRNA splicing upon the loss of USP39. Next, we calculated splicing efficiency using the RNA-seq data and found that knock down of USP39 resulted in significantly reduced 5′ and 3′ splice site efficiency (Fig. 6C). These data suggest that USP39 acts as a global splicing regulator of splicing accuracy and efficiency.

To systematically identify USP39-associated RNAs, RIP-seq was performed using an anti-FLAG antibody to precipitate RNAs from A2780 cells ectopically expressing FLAG-tagged USP39. The sequencing results showed that most of the USP39 binding sites mapped to introns (43.73%) and exons (28.52%) as illustrated in Fig. 6D–E and Table S7. To clarify the transcriptome-wide binding pattern of USP39, we analyzed regions flanking the 3′ and 5′ end of exons with USP39 crosslinks. Notably, the USP39 binding sites were highly enriched in exon-intron regions near both 3′ and 5′ splice sites (Fig. 6F). Next, we conducted GO analysis of 161 genes (eFDR < 0.05) encoding transcripts bound by USP39. Interestingly, transcription coactivator activity and hormone receptor binding pathways were highly enriched (Fig. 6G). Furthermore, we identified 11 candidate USP39-spliced transcripts from in a combined analysis of RIP-seq and RNA-seq data (Fig. 6H). Moreover, we validated four genes (HOXB6, SCAF11, KPNB1, and HMGA2) by RT-PCR and found that with USP39 knockdown, the levels of pre-mRNAs increased whereas the levels of spliced mRNAs decreased (Fig. 6I), indicating good reliability of RIP-seq and RNA-seq data.

### USP39 facilitates efficient splicing of the oncogenic transcription factor HMGA2

**Fig. 7:**
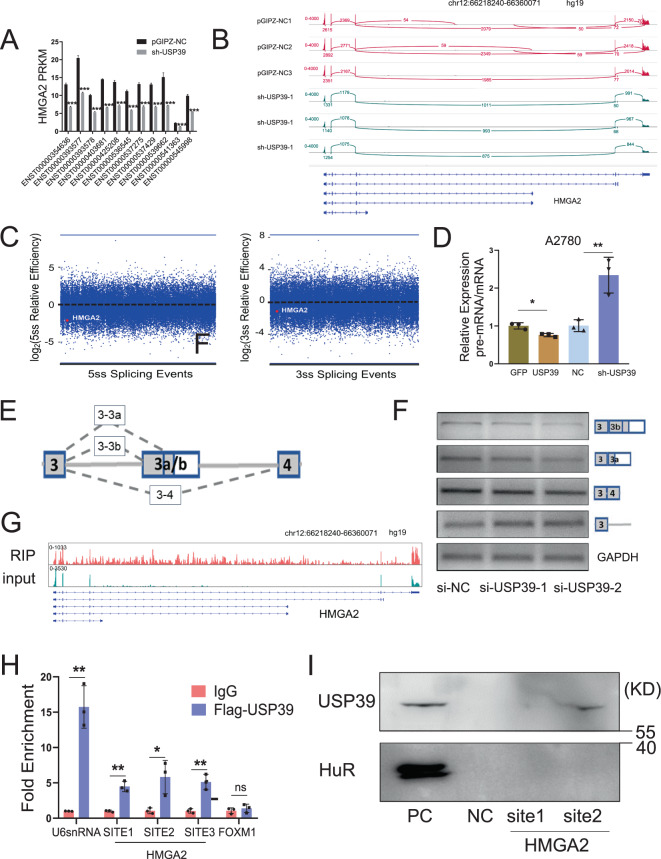
USP39 facilitates efficient splicing of the oncogenic transcription factor HMGA2. **A** Expression of individual HMGA2 transcript isoforms was analyzed using RNA-seq data for A2780 cells with USP39 knockdown (sh-USP39) and control cells (*n* = 3 biologically independent samples). **B** Sashimi plot visualization of RNA-seq reads mapping to HMGA2 in A2780 cells in response to USP39 knockdown. **C** Splicing efficiency analysis of HMGA2 at the 5′ and 3′ splicing sites upon USP39 depletion based on RNA-seq data. The red point represents HMGA2. Efficiency 5′ = transread count/ 5′ intron end first base coverage; Efficiency 3′ = transread count/ 3′ intron end last base coverage. **D** Pre-mRNA/mRNA ratio in A2780 cells with USP39 overexpression or knockdown (sh-USP39) was measured by q-PCR. **E** Schematic diagram showing HMGA2 minigene constructs and alternative splicing types. **F** The expression of HMGA2 minigene transcripts in Hela cells transfected HMGA2 minigenes in combination with USP39 siRNAs was measured by semi-quantitative PCR. **G** IGV tracks displaying the coverage of HMGA2 by RIP-seq reads. **H** The interaction between USP39 and the HMGA2 transcript was validated by RIP-PCR of A2780 cells overexpressing FLAG-USP39. FLAG antibody was used for immunoprecipitation. U6snRNA served as a positive control and FOXM1 as a negative control (*n* = 3 biologically independent samples). **I** RNA-pull down assay showing the interaction between the HMGA2 transcript and USP39 protein. HMGA2 site1 is an intronic sequence and the site2 sequence spanned an exon-intron junction. *P* value was obtained by Student’s *t*-test. Results represent the mean ± SD of three independent experiments. *P < 0.05, **P < 0.01, ***P < 0.001.

Among the validated USP39-regulated alternative splicing events, we next focused on HMGA2, an oncogenic transcription coactivator, which is commonly overexpressed in HGSOCs. We analyzed the differential expression of individual HMGA2 transcript isoforms using RNA-seq data. Unexpectedly, all eleven isoforms of HMGA2 were significantly downregulated in A2780 cells with knockdown of USP39 expression (Fig. 7A). Further analysis of sequencing reads that span exon boundaries clearly demonstrated a significant reduction in exon-exon junction reads near the 5′ and 3′ ends of the HMGA2 transcript upon USP39 depletion (Fig. 7B). Consistent with this, loss of USP39 led to decreased splicing efficiency of HMGA2 at both the 5′ and 3′ splice sites (Fig. 7C). To validate the USP39-regulated splicing of HMGA2, the ratio of pre-mRNA to mature mRNA was measured by RT-PCR with specific primers. In accordance with RNA-seq analysis, the amount of pre-mRNA splicing decreased when USP39 was depleted. On the contrary, the amount of pre-mRNA splicing increased with forced expression of USP39 (Fig. 7D). To gain more evidence, we constructed minigenes spanning alternative exons or introns (Fig. 7E). Splicing assays were conducted by transfecting cells with HMGA2 minigenes in combination with USP39 siRNAs. USP39 depletion markedly reduced the splicing of the three exon-exon minigene transcripts and increased splicing of the exon-intron minigene transcript (Fig. 7F). These results clearly indicate that USP39 is required for efficient splicing of HMGA2 pre-mRNA.

To determine whether USP39 plays a direct role in regulating HMGA2 splicing, we analyzed RIP-seq data. Importantly, there were abundant USP39 binding peaks on HMGA2 transcripts (Fig. 7G). To further validate the RIP-seq peaks, we performed RIP-PCR and found that USP39 bound to three sites in the HMGA2 transcripts (Fig. 7H). Consistent with this, USP39 bound to biotin-labeled HMGA2 in an RNA pull-down assay (Fig. 7I), indicating a direct interaction between USP39 and HMGA2. Moreover, we conducted ChIP-seq analysis of A2780 cells using anti-FLAG and anti-POLR2A antibodies, and found that USP39 was preferentially bound to intronic and intergenic regions, but not the promoter region which is bound by RNA polymerase II (Supplementary Fig. 4a). These findings indicate that USP39 regulates HMGA2 expression through alternative splicing but not transcription initiation.

### USP39 promotes tumor progression by increasing HMGA2 levels in ovarian cancer cells

**Fig. 8:**
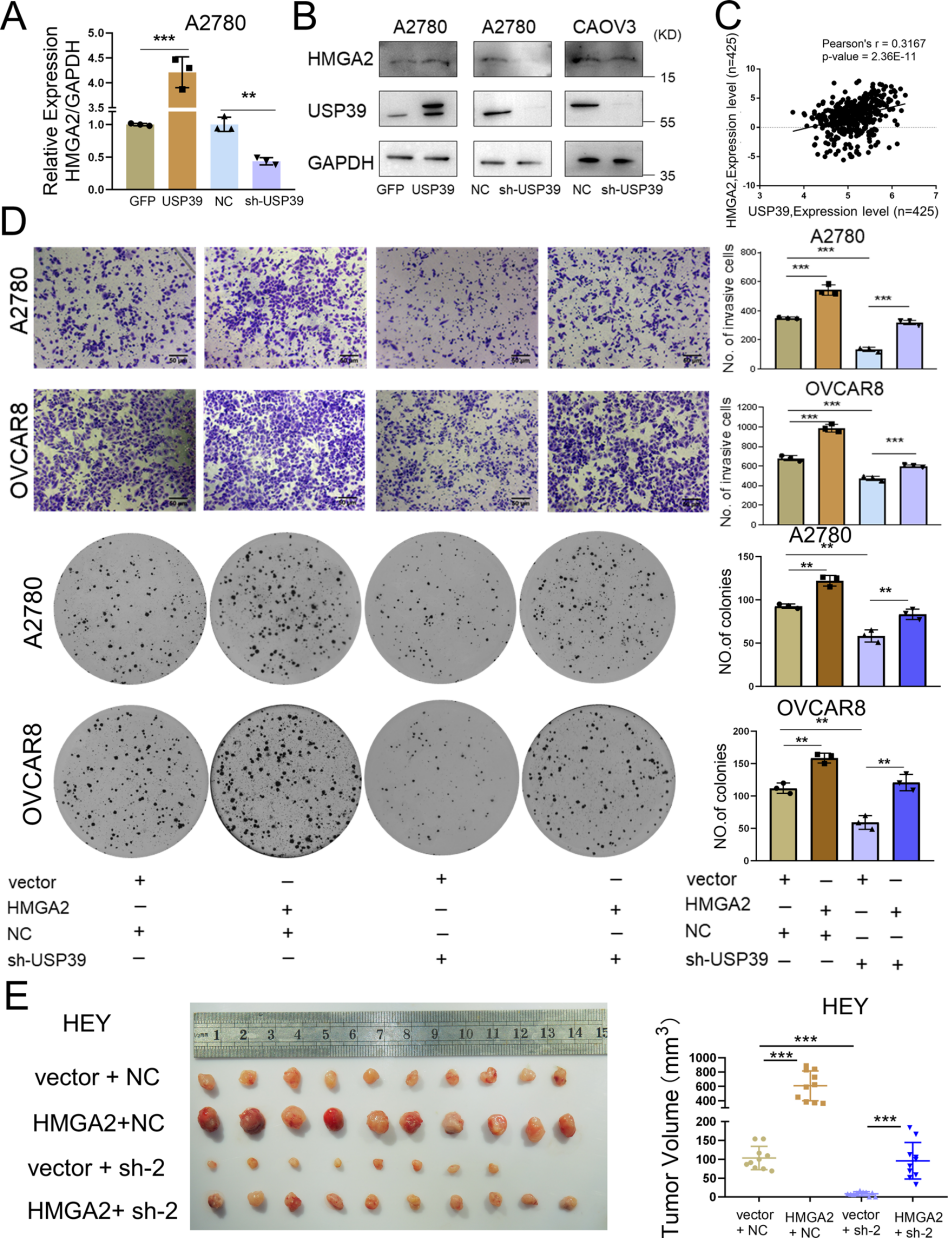
USP39 promotes tumor progression by increasing HMGA2 levels in ovarian cancer cells. **A** Relative HMGA2 mRNA expression as measured by qPCR in A2780 cells with USP39 overexpression (USP39) or knockdown (sh-USP39) compared to corresponding control (*n* = 3 biologically independent samples). **B** Immunoblot analysis of HMGA2 and USP39 protein levels in ovarian cancer cells with USP39 overexpression or knockdown. **C** Pearson correlation analysis of USP39 and HMGA2 expression in HGSOCs from the TCGA cohort. **D** Transwell and clonogenic assays for investigating the potential of HMGA2 to rescue the loss of USP39 in ovarian cancer cells as indicated (*n* = 3 biologically independent samples). **E** Xenograft experiments showed HMGA2 could rescue the loss of USP39 in tumor growth in vivo (*n* = 10 mice per group). *P* value was obtained by Student’s *t*-test and ANOVA analysis. Results represent the mean ± SD of three independent experiments. *P < 0.05, **P < 0.01, ***P < 0.001.

We next investigated the functional significance of the USP39-HMGA2 axis. We first measured HMGA2 expression in A2780 and CAOV3 ovarian cancer cell lines with USP39 knockdown and in A2780 cells with USP39 overexpression. Ectopic expression of USP39 increased the mRNA and protein levels of HMGA2, whereas USP39 knockdown had the opposite effect (Fig. 8A–B). Next, we analyzed TCGA expression data and found that HMGA2 was positively correlated with USP39 (Fig. 8C). Pan-cancer analysis showed that HMGA2 was upregulated in various types of cancer (Supplementary Fig. 5A). Importantly, high levels of HMGA2 indicated poor overall survival and progression-free survival of ovarian cancer patients according to the data from the Kaplan–Meier Plotter (Supplementary Fig. 5B). We then speculated that HMGA2 might increase the oncogenic capacity of ovarian cancer cells. Consistent with this hypothesis, ectopic expression of HMGA2 significantly enhanced the invasive and clonogenic capacity of A2780 and OVCAR8 cells and xenograft growth of HEY cell (n=10 per group) (Fig. 8D–E). In addition, overexpression of HMGA2 rescued the inhibition of proliferation, invasion and xenograft growth induced by silencing USP39 (Fig. 8D–E). These results indicate that USP39 facilitates oncogenic potential partially through regulating HMGA2.

## Discussion

Splicing factors can act as both oncoproteins and tumor suppressors^24^. Previous studies have shown that USP39 is commonly upregulated in various human cancers including ovarian cancer^25,26^. High USP39 expression is correlated with poor prognosis in patients with prostate and gastric cancers^27,28^. In this study, we demonstrate for the first time that high expression of USP39 indicated poor prognosis in HGSOCs both in our cohort and TCGA cohort. Clinicopathologic analysis revealed that USP39 overexpression was associated with advanced tumor stage and platinum resistance (Supplementary Table 5). Subsequent functional experiments revealed that USP39 promoted the proliferation and invasion of ovarian cancer cells in vitro (Fig. 2) and tumor growth in vivo (Fig. 3). These findings indicate that USP39 has an oncogenic function and could be a potential target for ovarian cancer therapy.

Fidelity and efficiency of pre-mRNA splicing are critical for generating functional mRNAs^29^. Previously, USP39 depletion was found to cause a significant reduction in pre-mRNA splicing efficiency in colon cancer and glioma^25,30^. However, how USP39 affects alternative splicing and its targets have not been fully elucidated. To define genome-wide USP39-RNA interactions and identify alternative splicing events regulated by USP39, we performed RNA-seq and RIP-seq approaches. RNA-sequencing analysis revealed that USP39 depletion led to globally impaired splicing that is characterized by skipped exons and overrepresentation of introns and intergenic regions. RIP-seq data analysis showed that USP39 was highly enriched in exon-intron regions near both the 5′ and 3′ splice sites (Fig. 6E–F). These data suggest that USP39 acts as a global regulator of splicing accuracy and efficiency.

Combined analysis of RIP-seq and RNA-seq data, we identified USP39 facilitates efficient splicing of HMGA2 and that USP39 exerts oncogenic potential partially through regulating HMGA2 in ovarian cancer cells (Fig. 7). HMGA2 is a non-histone chromatin factor that is highly expressed in fetal tissue and malignant tumors^31^. We previously reported that HMGA2 was frequently overexpressed in HGSOC and that a high level of HMGA2 was significantly associated with poor prognosis in patients with HGSOC^32^. HMGA2 was identified as the target gene of several microRNAs including Let-7, and overexpression of HMGA2 can occur as a result of a shortened 3’UTR that lacks let-7 binding sites^33^. Splicing kinase CLK3 stimulates exon skipping of HMGA2 to generate a short isoform which promotes hematopoietic stem cell self-renewal^34^. Surprisingly, our work here demonstrated that USP39 improved splicing efficiency of HMGA2 rather than exon skipping. Together, these data revealed that USP39 is a core splicing factor that regulates alternative splicing events by modulating the recognition or cleavage of 5′ and 3′ splice sites and increasing splicing efficiency.

Transcription and pre-mRNA splicing are tightly coupled gene expression events in eukaryotic cells^35^. SC35 is part of the 7SK complex assembled at gene promoters and plays a direct role in transcription pause release^36^. HNRNPL and RBM25 directly participate in transcription through functional interaction with specific transcription factors^37^. SKIP plays a role in transcription elongation through binding to P-TEFb^38^. Our ChIP-seq data revealed that USP39 was preferentially bound to intronic and intergenic regions. In comparison to splicing factors that bind to promoter regions such as SC35 and RBM25, USP39 might not participate in transcription initiation. Whether USP39 is involved in transcription elongation requires further investigation.

Our work also provides evidence for the upregulation of USP39 in ovarian cancer. We analyzed the USP39 promoter region to predict potential transcription factors that drive USP39 expression. Strikingly, c-MYC was predicted to bind the USP39 promoter. We subsequently performed luciferase reporter and ChIP assays and identified c-MYC as a direct regulator of USP39 transcription. HGSOC is characterized by extensive copy number alterations, among which the amplification of c-MYC occurs in nearly half of tumors^39^. c-MYC directly upregulates core splicing machinery genes, including PRMT5^40^. c-MYC was also reported to drive the transcription of SRSF1, sam68, PTB1, and several hnRNPs^41^. Importantly, splicing factor BUD31 is a c-MYC synthetic lethal gene^42^. Our findings here support the notion that c-MYC driven increases in transcription leave tumors dependent on reprograming of alternative splicing for survival.

Taken together, our study demonstrated that USP39 serves as an oncogenic factor in HGSOC by promoting splicing efficiency and accuracy. We also provided strong evidence that USP39 interacts with spliceosome components. In particular, USP39 facilitates efficient splicing of HMGA2 at both the 5′ and 3′ splice sites. Notably, c-MYC contributes to the regulation of USP39 transcription in ovarian cancer. USP39 could represent a novel prognostic biomarker and a potential target in HGSOC.

## Supplementary information

Supplementary Figures

Supplementary Table 1

Supplementary Table 2

Supplementary Table 3

Supplementary Table 4

Supplementary Table 5

Supplementary Table 6

Supplementary Table 7

Supplementary Western Blot
