# Supplementary material for: Splicing factor USP39 promotes ovarian cancer malignancy through maintaining efficient splicing of oncogenic HMGA2
Source: Cell Death Dis. 2021 Mar 17;12(4):294. doi: 10.1038/s41419-021-03581-3 (PMC7969951; doi:10.1038/s41419-021-03581-3)
Supplement: Supplementary file 1 — Supplementary Figures [file 41419_2021_3581_MOESM1_ESM.docx]

**Supplementary Figure S1**


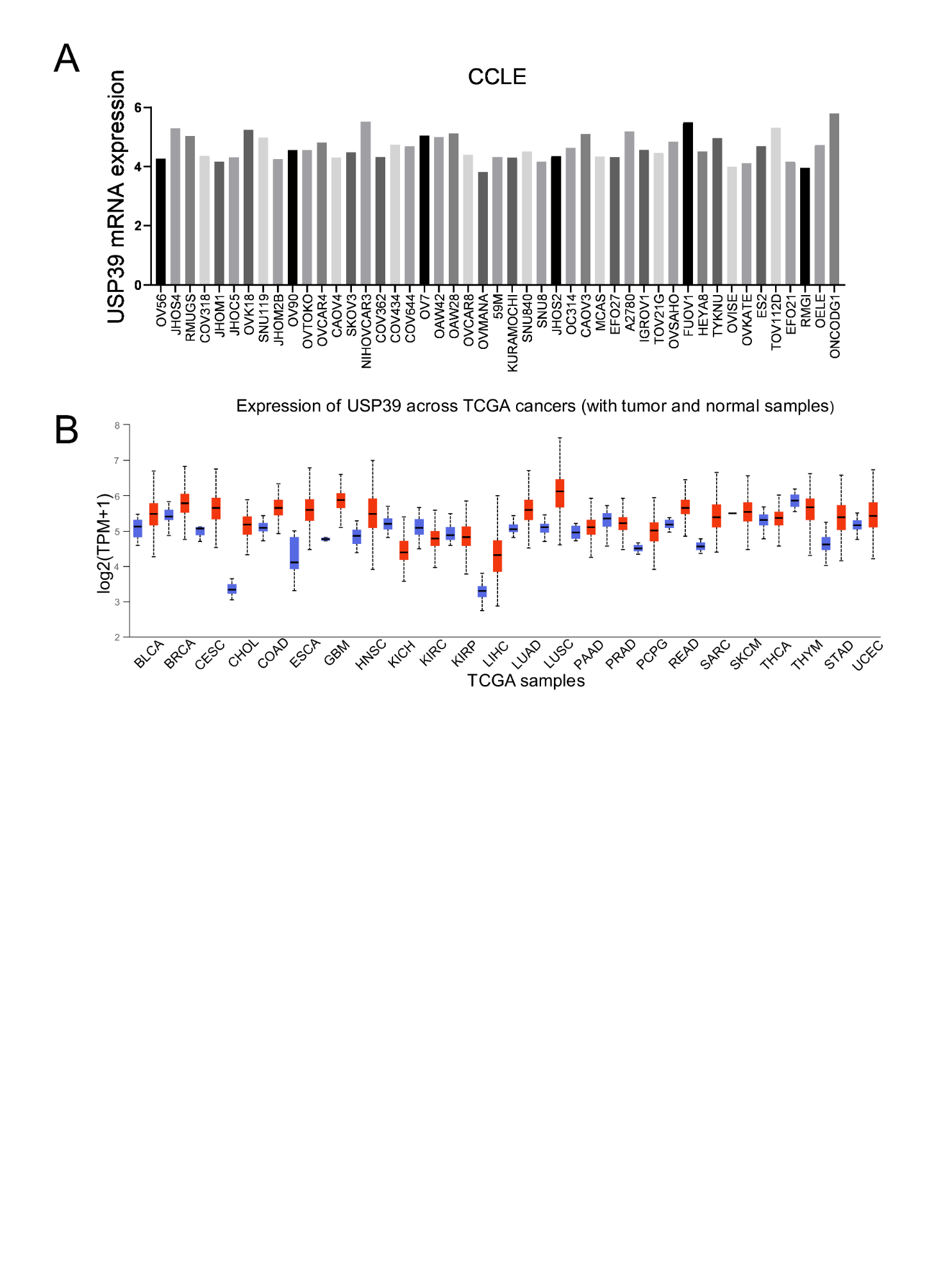


Supplementary Figure S1. Pan-cancer analysis showed that USP39 was upregulated in various cancer types (UALCAN).

**Supplementary Figure S2**


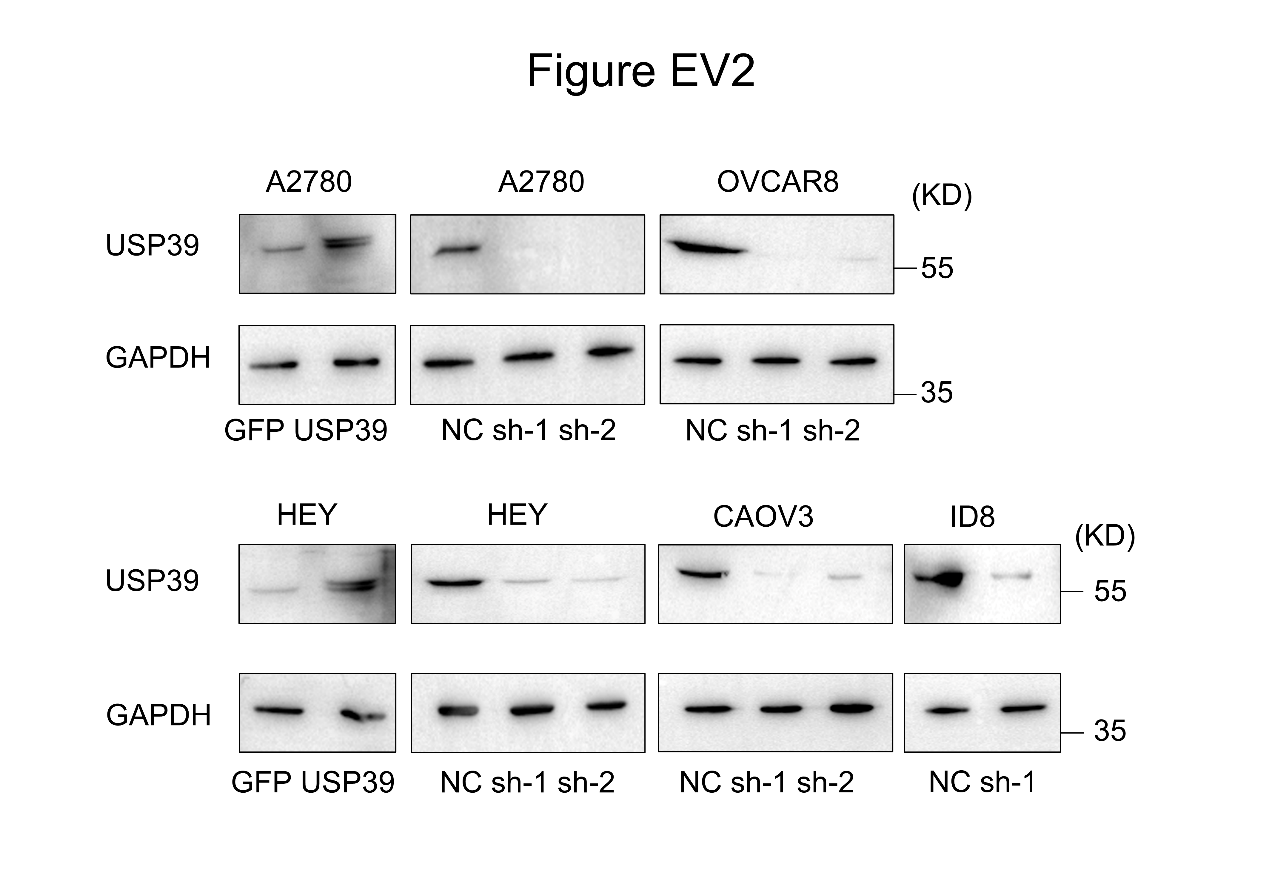


Supplementary Figure S2. USP39 overexpression and knockdown were confirmed by western blot in all ovarian cancer cell lines used in the research.

**Supplementary Figure S3**


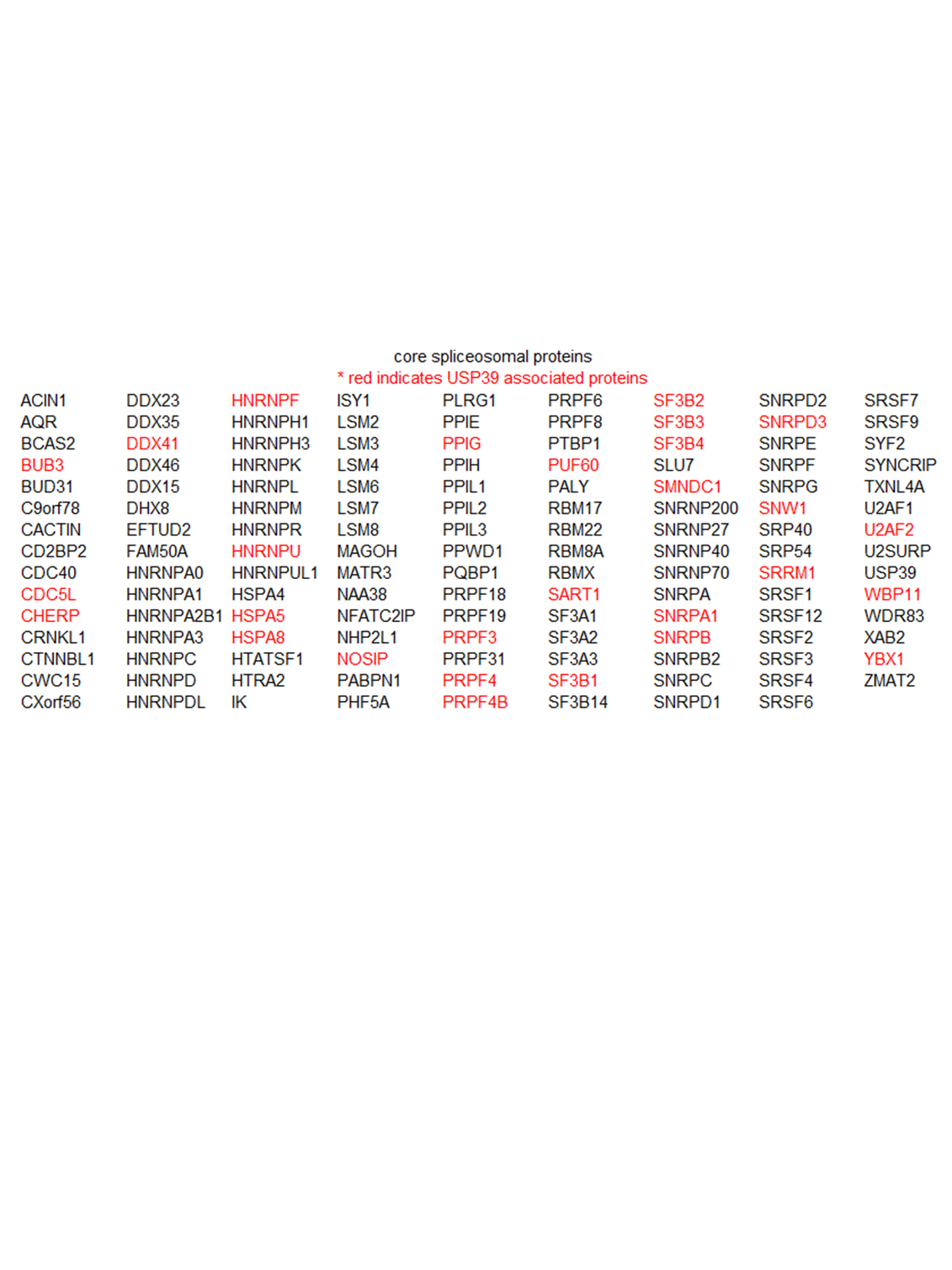


Supplementary Figure S3. 134 core spliceosomal proteins are listed. Proteins in red are shown to interact with USP39, as discovered by Flag-USP39 IP-MS.

**Supplementary Figure S4**


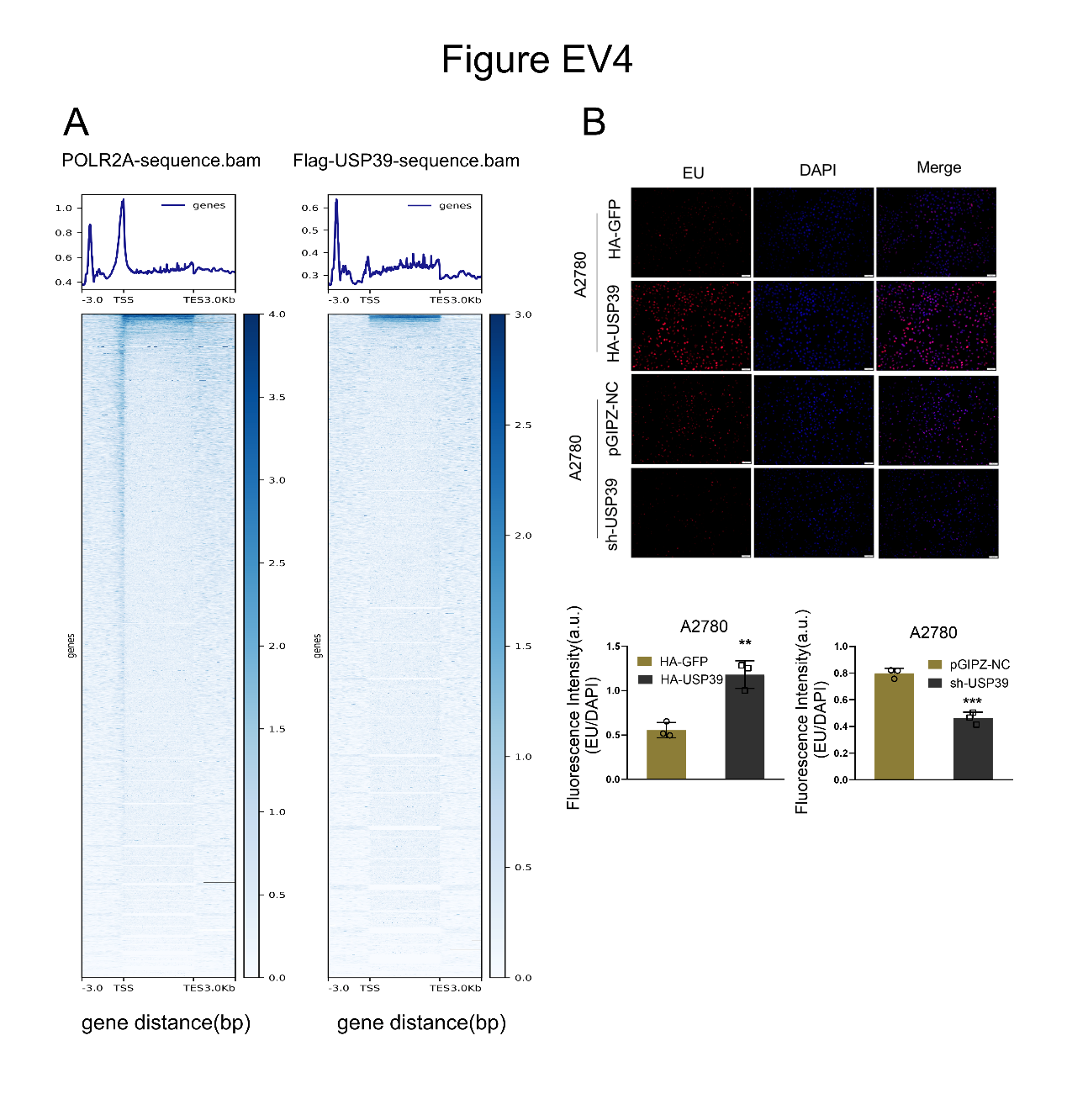


Supplementary Figure S4. (**A**) Metagene analysis of POLR2A and Flag-USP39 CHIP-seq reads signal distribution on gene body.

**Supplementary Figure S5**


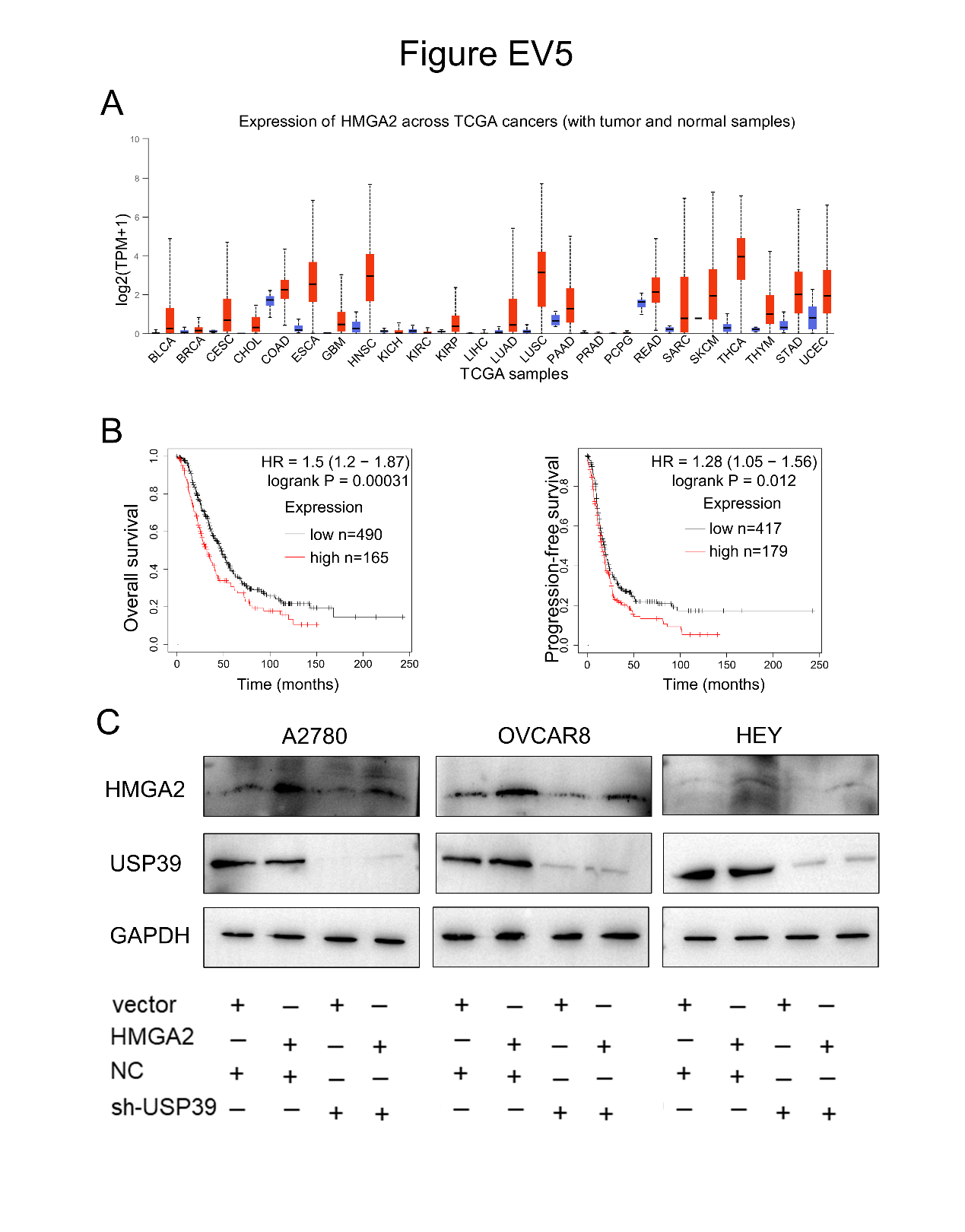


Supplementary Figure S5. (**A**) Pan-cancer analysis showed that HMGA2 was upregulated in various cancer types (UALCAN). (B) Kaplan–Meier analysis of the effect of HMGA2 expression on the overall survival and progression free survival of ovarian cancer patients (<http://kmplot.com/>). (C) rescue of HMGA2 expression in A2780, OVCAR3 and HEY cells were confirmed by western blot.
