# Supplementary material for: Splicing factor USP39 promotes ovarian cancer malignancy through maintaining efficient splicing of oncogenic HMGA2
Source: Cell Death Dis. 2021 Mar 17;12(4):294. doi: 10.1038/s41419-021-03581-3 (PMC7969951; doi:10.1038/s41419-021-03581-3)
Supplement: Supplementary file 4 — Supplementary Table 3 [file 41419_2021_3581_MOESM4_ESM.docx]

| method | name | sequence(5 ′—3 ′) |
| --- | --- | --- |
| si-RNA | si-USP39-1 | TCAAGAGATTCACTAAGAA |
| si-RNA | si-USP39-2 | GCAGTTGTACTTTGCAGTA |
| si-RNA | negative control | TTCTCCGAACGTGTCACGT |
| sh-RNA | sh-USP39-1 | CCGGGATTTGGAAGAGGCGAGATAACTCGAG  TTATCTCGCCTCTTCCAAATCTTTTTG |
| sh-RNA | sh-USP39-2 | CCGGGTTGCCTCCATATCTAATCTTCTCGAGAAG  ATTAGATATGGAGGCAACTTTTT |
| sh-RNA | sh-c-MYC | CCGGCCTGAGACAGATCAGCAACAACTCGAGT  TGTTGCTGATCTGTCTCAGGTTTTTG |
| qPCR | GAPDH-F | CAGAACATCATCCCTGCCTCTAC |
| qPCR | GAPDH-R | TTGAAGTCAGAGGAGACCACCTG |
| qPCR | USP39-F | CACTTACCTGCCGGGTATTGT |
| qPCR | USP39-R | CCTGGAGGACGTTTGATGTTCT |
| qPCR | HMGA2-mRNA-F | ACCCAGGGGAAGACCCAAA |
| qPCR | HMGA2-mRNA-R | CCTCTTGGCCGTTTTTCTCCA |
| qPCR | HMGA2-preRNA-F | CACAGTGTGGCTATGGTGTG |
| qPCR | HMGA2-preRNA-R | CCATCTGAAGCTGGGAATGA |
| qPCR | HMGA2-ex1-int1-F | CAGCAGCAAGTCAGTACGAG |
| qPCR | HMGA2-ex1-int1-R | GCACAATAGCGAAAGTCCGA |
| qPCR | HMGA2-int4-ex5-F | ATCCTCTTTTGAGGCAAGCA |
| qPCR | HMGA2-int4-ex5-R | TCTTCGGCAGACTCTTGTGA |
| RT-PCR | HMGA2-minigene-3-4-F | AAAGCAGAAGCCACTGGAGAAAAAC |
| RT-PCR | HMGA2-minigene-3-4-R | CTGAGCAGGCTTCTTCTGAACAACT |
| RT-PCR | HMGA2-minigene-3-3a/b-F | AAAGCAGAAGCCACTGGAGAAAAAC |
| RT-PCR | HMGA2-minigene-3-3a/b-R | TCAGCCCACCCTCTACTCTCTAAGC |
| RT-PCR | HMGA2-minigene-3-int-F | AAAGCAGAAGCCACTGGAGAAAAAC |
| RT-PCR | HMGA2-minigene-3-int-R | TGCACCCACTAACTCGTCAT |
| RIP-PCR | U6snRNA-F | CTCGCTTCGGCAGCACA |
| RIP-PCR | U6snRNA-R | AACGCTTCACGAATTTGCGT |
| RIP-PCR | HMGA2 site1-F | TGTGCACATGTACCCTAAAACT |
| RIP-PCR | HMGA2 site1-R | AATTAGCTGGGTGTGGTGGT |
| RIP-PCR | HMGA2 site2-F | CCATCGTGCCTGGTCTAAAA |
| RIP-PCR | HMGA2 site2-R | TGAAGAGCTATCCTGGACTCC |
| RIP-PCR | HMGA2 site3-F | CACAGTGTGGCTATGGTGTG |
| RIP-PCR | HMGA2 site3-R | CCATCTGAAGCTGGGAATGA |
| RIP-PCR | FOXM1 | TTCTCTCCCACAATGCCTGG |
| RIP-PCR | FOXM1 | CTCTGTCCACTAAAGCCATGC |
| RT-PCR | HOXB6-mRNA-F | CAACAGTTCCTCCTTTGGGC |
| RT-PCR | HOXB6-mRNA-R | TTTTCCACTTCATGCGTCGG |
| RT-PCR | HOXB6-preRNA-F | CAGTGAGTGAGACTTCCCGT |
| RT-PCR | HOXB6-preRNA-R | AATAGGGGACACAGCGAGAG |
| RT-PCR | KPNB1-mRNA-F | TTCAGAGGCAGCAGAACAAG |
| RT-PCR | KPNB1-mRNA-R | ATGAAGGGGAGGACATGTGG |
| RT-PCR | KPNB1-preRNA-F | GGTGAGCTGGGGAGAACTAT |
| RT-PCR | KPNB1-preRNA-R | GCTTGGCAATAACGTGAGC |
| RT-PCR | SCAF11-mRNA-F | TGGAAGGTGAAGAAAACGGAG |
| RT-PCR | SCAF11-mRNA-R | AGCTTTCTGGAAAACCAACTTC |
| RT-PCR | SCAF11-preRNA-F | TCCAAACTGTCCACTTGTATACA |
| RT-PCR | SCAF11-preRNA-R | CAGAGGACAGTACCAAACGCT |
| ChIP-PCR | USP39-F | ACAAATTTTCTGCAGCGTGA |
| ChIP-PCR | USP39-R | GCCGGACATCTCCACTACC |
| ChIP-PCR | 16q22-F | CTACTCACTTATCCATCCAGGCTAC |
| ChIP-PCR | 16q22-R | ATTTCACACACTCAGACATCACAG |
