# Supplementary material for: Splicing factor USP39 promotes ovarian cancer malignancy through maintaining efficient splicing of oncogenic HMGA2
Source: Cell Death Dis. 2021 Mar 17;12(4):294. doi: 10.1038/s41419-021-03581-3 (PMC7969951; doi:10.1038/s41419-021-03581-3)
Supplement: Supplementary file 5 — Supplementary Table 4 [file 41419_2021_3581_MOESM5_ESM.docx]

**Supplementary Table 3**

Primary antibody information

| Antibody | Company | Catalog number |
| --- | --- | --- |
| USP39 | Abcam | ab131244 |
| E-Cadherin | ABclonal | A3044 |
| N-Cadherin | ABclonal | A3035 |
| β-Catenin | CST | 8480 |
| Vimentin | CST | 5741 |
| Snail | ABclonal | A5243 |
| Slug | ABclonal | A1057 |
| U2AF2 | Santa Cruz | sc-53942 |
| PRPF3 | ABclonal | A5482 |
| SART1 | ABclonal | A8569 |
| SNRPA1 | ABclonal | A12161 |
| p-SF3B1 | CST | 25009 |
| SF3B1 | Proteintech | 27684-1-AP |
| SC35 | Abcam | ab204916 |
| Flag tag | CST | 14793 |
| HMGA2 | CST | 8179 |
| POLR2A | Sigma-Aldrich | 05-623 |
| c-MYC | CST | 9402 |
| HuR | Abcam | ab136542 |
| GAPDH | Proteintech | 60004-1-Ig |
