# Supplementary material for: Splicing factor USP39 promotes ovarian cancer malignancy through maintaining efficient splicing of oncogenic HMGA2
Source: Cell Death Dis. 2021 Mar 17;12(4):294. doi: 10.1038/s41419-021-03581-3 (PMC7969951; doi:10.1038/s41419-021-03581-3)
Supplement: Supplementary file 6 — Supplementary Table 5 [file 41419_2021_3581_MOESM6_ESM.docx]

| **Table S5**  Correlation between USP39 expression and clinicopathological characteristics | | | |
| --- | --- | --- | --- |
| **Clinicopathological Feature** | **Number** | **USP39 expression**  **Low High** | ***P* value ^a^** |
| **Age (years)** |  |  |  |
| <60 | 102 | 70 32 | 0.1148 |
| ≥60 | 47 | 26 21 |  |
| **FIGO Staging** |  |  |  |
| Ⅰ-Ⅱ | 35 | 28 7 | 0.0278 |
| Ⅲ-Ⅳ | 114 | 68 46 |  |
| **CA 125 in serum (U/mL)** |  |  |  |
| <500 | 48 | 29 19 | 0.6073 |
| ≥500 | 91 | 59 32 |  |
| **Platinum status** | | | |
| Sensitive | 38 | 31 7 | 0.0228 |
| Resistance | 14 | 7 7 |  |
| **Lymph node metastasis** |  |  |  |
| Absent | 29 | 19 10 | 0.8415 |
| Present | 22 | 15 7 |  |
| **Omentum metastasis** |  |  |  |
| Absent | 33 | 23 10 | 0.4464 |
| Present | 101 | 63 38 |  |
