# Supplementary material for: Splicing factor USP39 promotes ovarian cancer malignancy through maintaining efficient splicing of oncogenic HMGA2
Source: Cell Death Dis. 2021 Mar 17;12(4):294. doi: 10.1038/s41419-021-03581-3 (PMC7969951; doi:10.1038/s41419-021-03581-3)

Figure 2

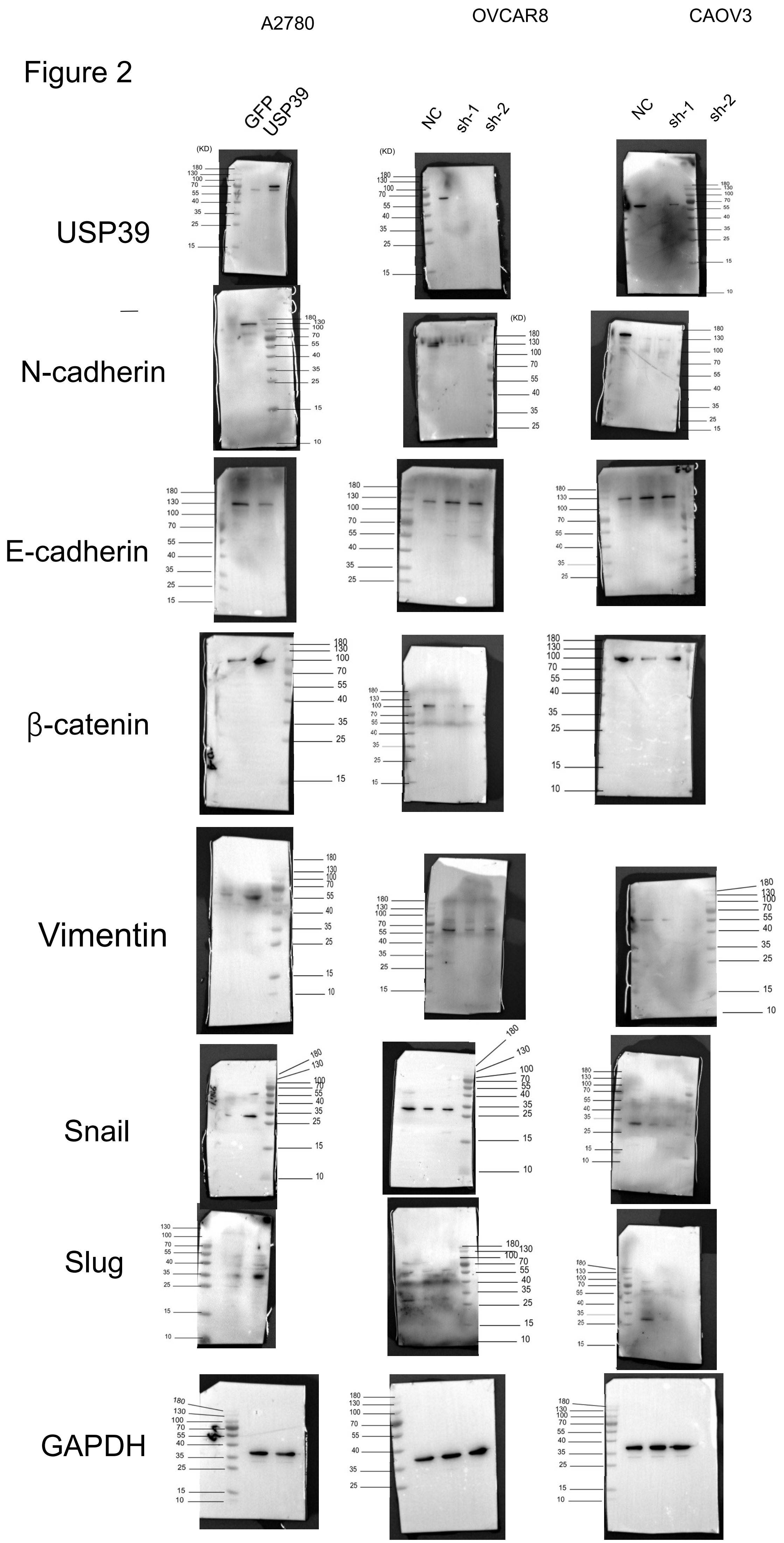
A2780
OVCAR8
CAOV3
GFP
USP39
NC
sh-1
sh-2
USP39
N-cadherin
E-cadherin
β-catenin
Vimentin
Snail
Slug
GAPDH
(KD)
180
130
100
70
55
40
35
25
15
10

Figure 4

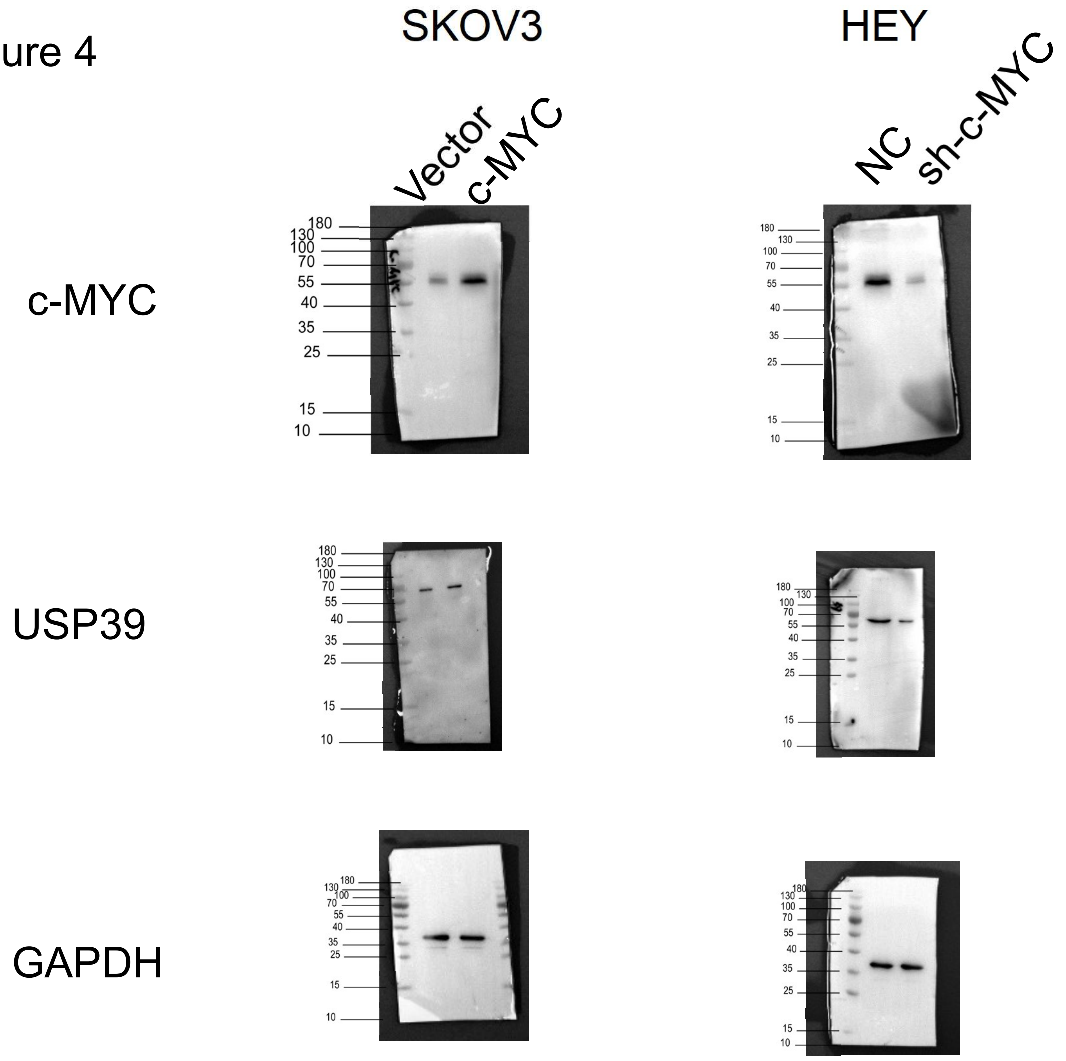
SKOV3
HEY
Vector
c-MYC
NC
sh-c-MYC
c-MYC
USP39
GAPDH
180
130
100
70
55
40
35
25
15
10

Fig 5

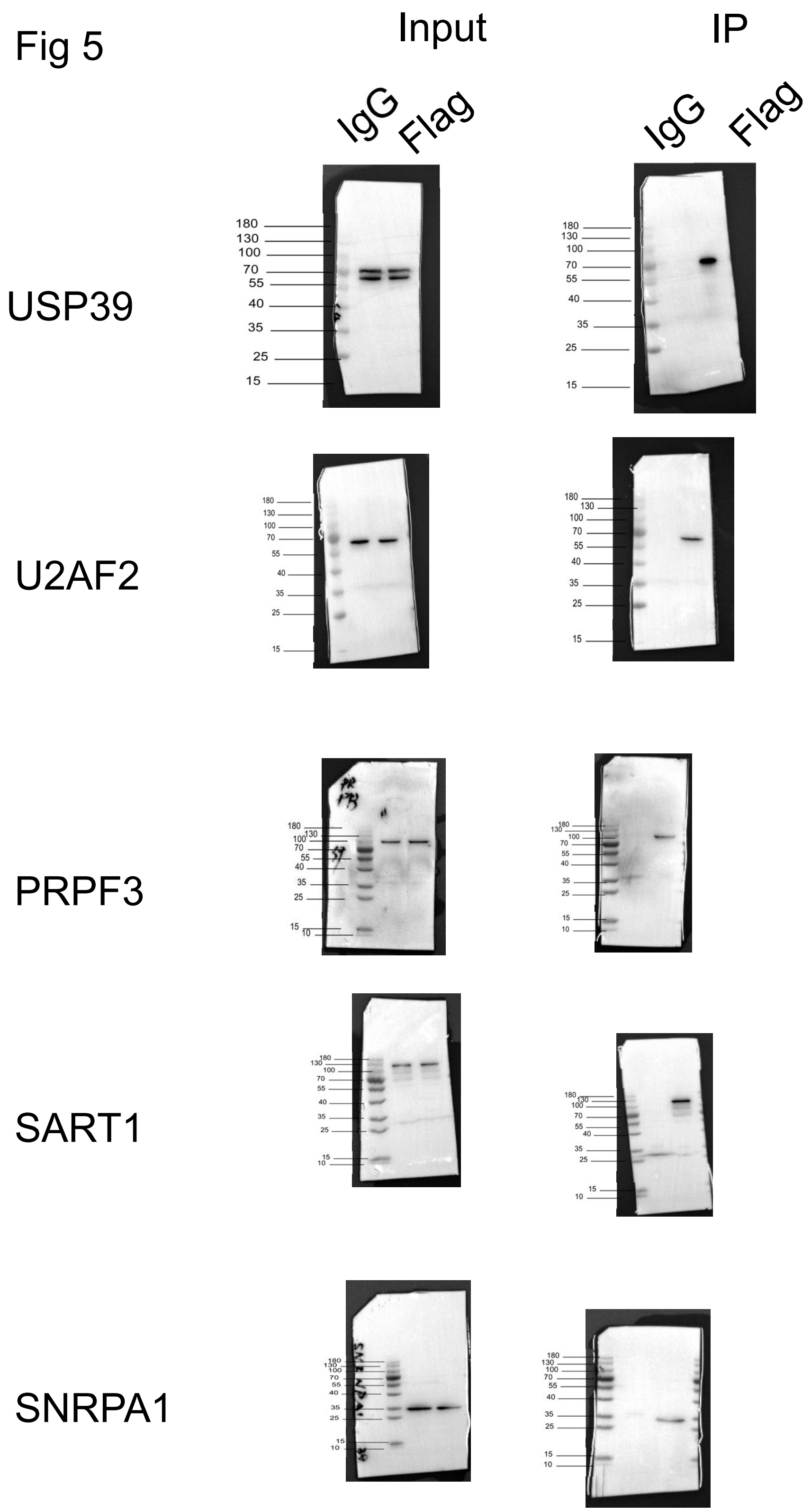
Input
IP
IgG
Flag
IgG
Flag
USP39
U2AF2
PRPF3
SART1
SNRPA1
180
130
100
70
55
40
35
25
15
10

Fig 5

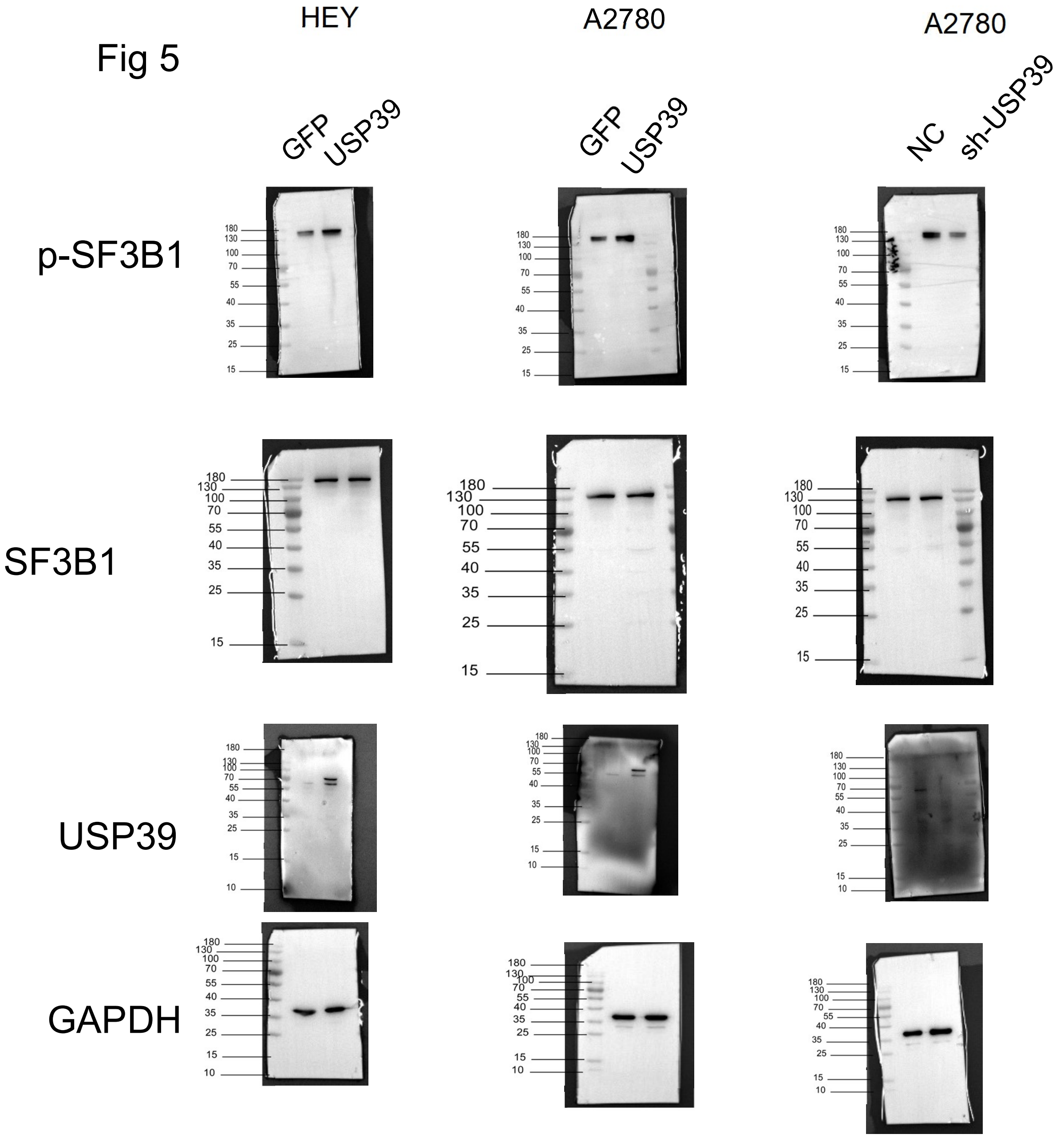
HEY
A2780
A2780
GFP
USP39
GFP
USP39
NC
sh-USP39
p-SF3B1
SF3B1
USP39
GAPDH
180
130
100
70
55
40
35
25
15
10

Fig 7

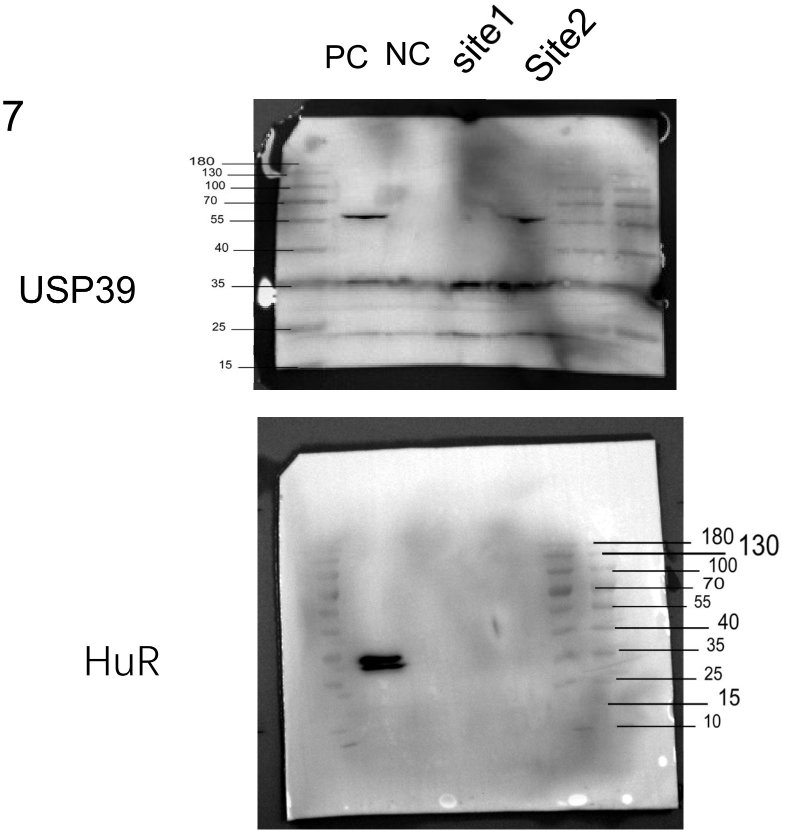
PC
NC
site1
Site2
USP39
HuR
180
130
100
70
55
40
35
25
15
10

Fig 8

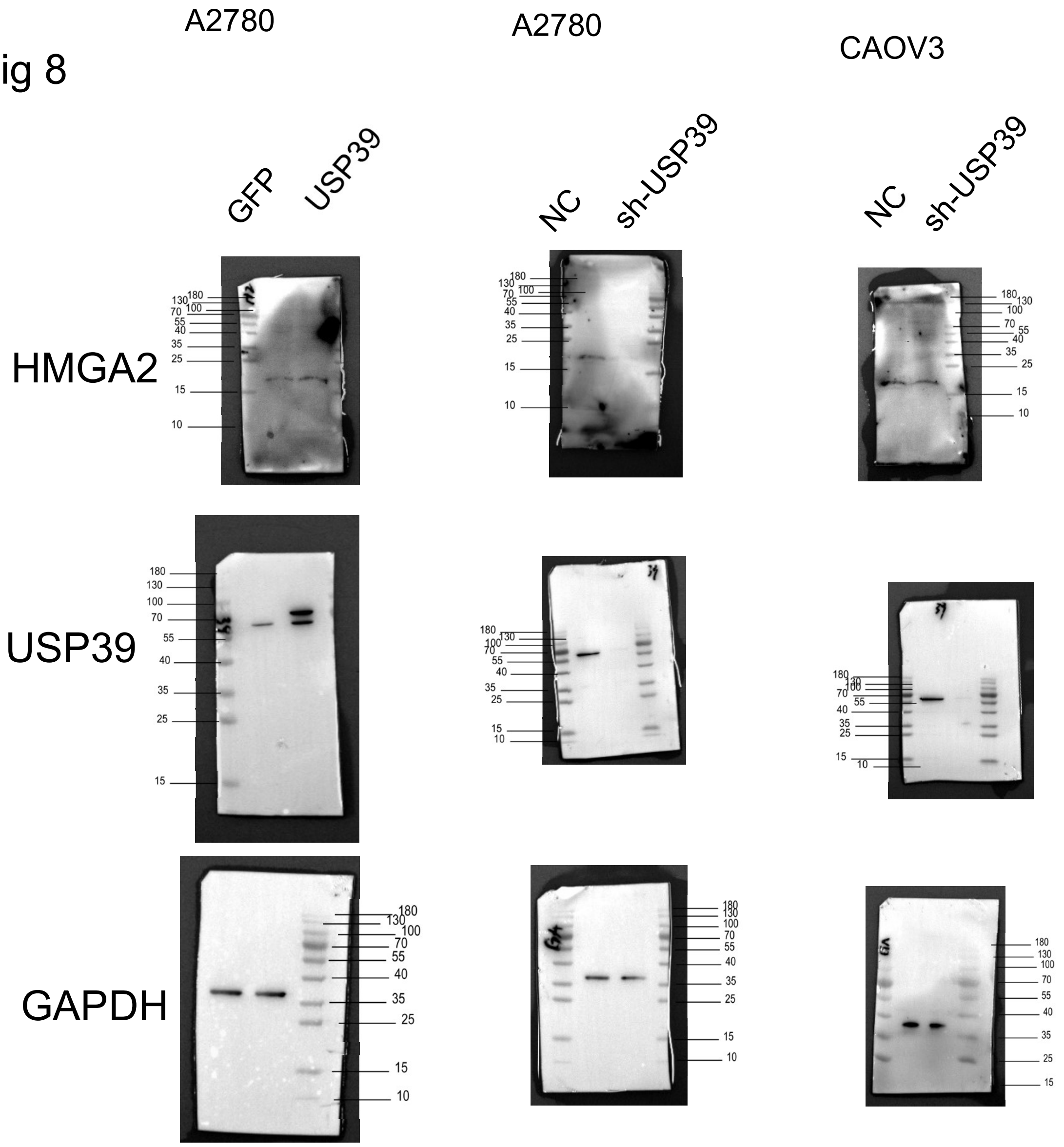
A2780
A2780
CAOV3
GFP
USP39
NC
sh-USP39
NC
sh-USP39
HMGA2
USP39
GAPDH
180
130
100
70
55
40
35
25
15
10

Supplementary Fig. 2

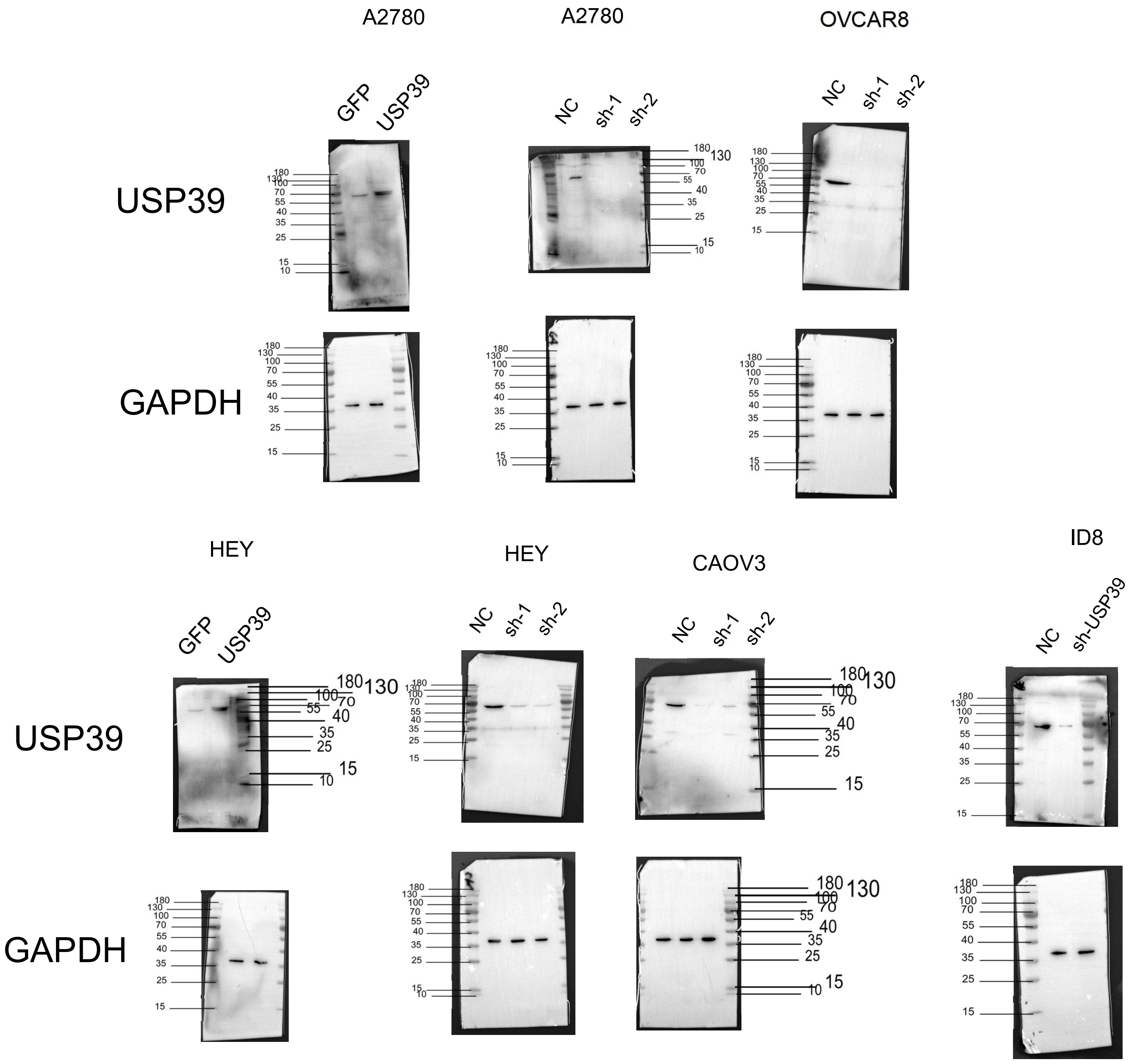

Supplementary Fig. 5

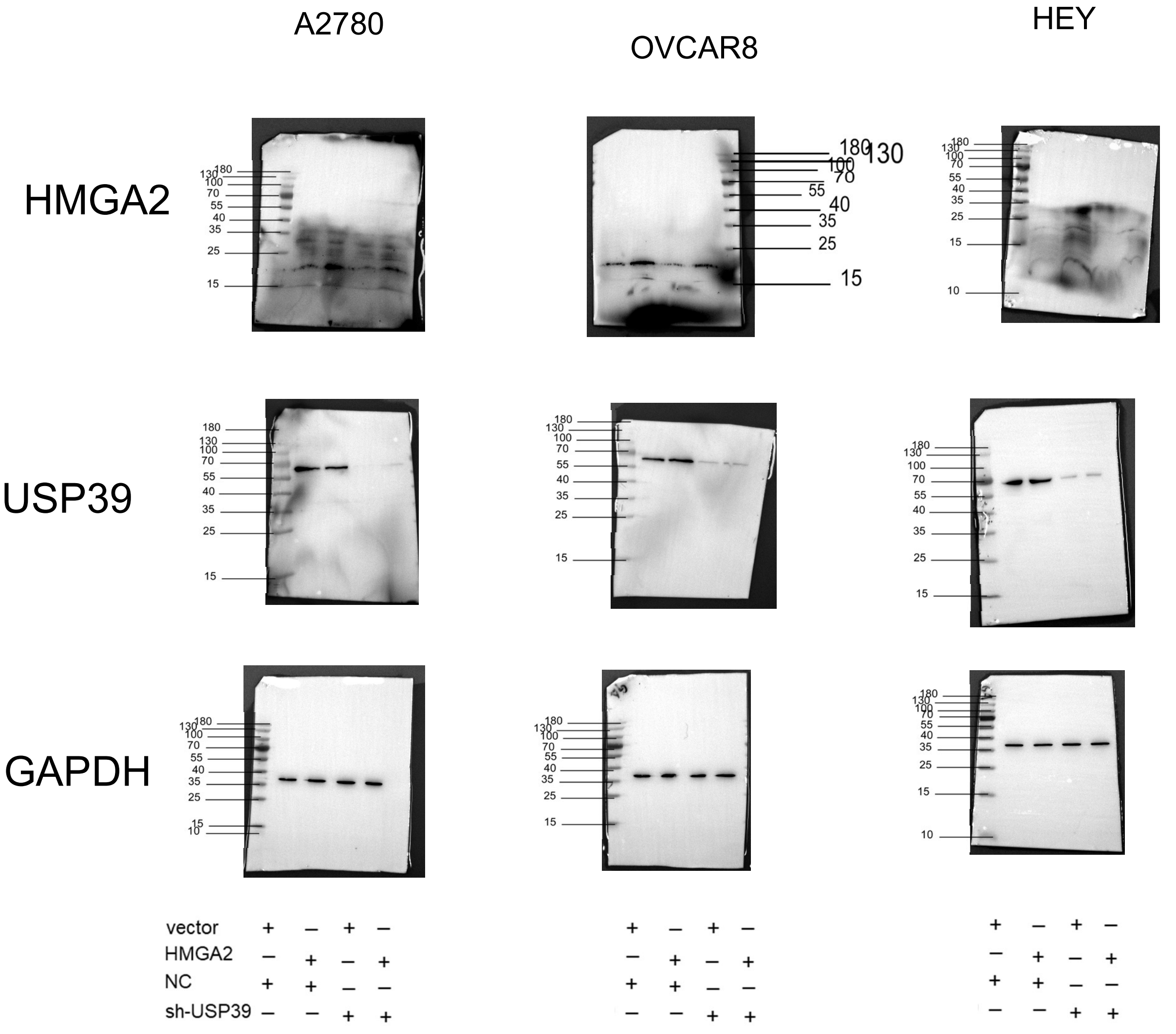

Supplement: Supplementary file 9 — Supplementary Western Blot [file 41419_2021_3581_MOESM9_ESM.pdf]
